# Supplementary material for: Does melatonin administration reduce the incidence of postoperative delirium in adults? Systematic review and meta-analysis
Source: BMJ Open. 2023 Mar 29;13(3):e069950. doi: 10.1136/bmjopen-2022-069950 (PMC10069576; doi:10.1136/bmjopen-2022-069950)

Appendix 1: Search Strategy

[melatonin(MH) OR ramelteon(TW) OR rozerem(TW) OR “melatonin agonist*”(TW) OR “N-Acetyl-5-methoxytryptamine”(TW) OR melatonin*(TW) OR Melatoniin*(TW) OR circadin(TW) OR “HT 903”(TW) OR melapure(TW) OR armonia(TW) OR melamil(TW) OR benedorm(TW) OR “BP 2013”(TW) OR BP2013 OR JL5DK93RCL(TW)]

AND

[delirium(MH) OR “emergence delirium”(MH), OR delirium(TW) OR “perioperative delirium”(TW) OR “postoperative delirium”(TW) OR “organic brain syndrome”(TW) OR “acute confusion”(TW)]

Dates: 1st January 1990 – 5th April 2022

Other limits: Nil

CINAHL Search Strategy

#	Query
S24	S16 AND S22
S23	S16 AND S22
S22	S17 OR S18 OR S19 OR S20 OR S21
S21	((postoperative or perioperative) N1 delirium)
S20	"acute confusion"
S19	"organic brain syndrome"
S18	Delirium
S17	(MH "Delirium")
S16	S1 OR S2 OR S3 OR S4 OR S5 OR S6 OR S7 OR S8 OR S9 OR S10 OR S11 OR S12 OR S13 OR S14 OR S15
S15	"JL5DK93RCL"
S14	"bp2013"
S13	"bp 2013"
S12	benidorm
S11	melamil
S10	armenia
S9	melapure
S8	"ht 903"
S7	circadian
S6	melatoniin*
S5	"n-acetyl-5-methoxytryptamine"
S4	rozerem
S3	ramelteon
S2	melatonin*
S1	(MM "Melatonin")

Dates – 1/1/1990 – 5/4/2022

Ovid Medline All and Embase

1.	**exp Melatonin/**
2.	melatonin*.mp.
3.	ramelteon.mp.
4.	rozerem.mp.
5.	"n-acetyl-5-methoxytryptamine".mp.
6.	melatoniin*.mp.
7.	circadian.mp.
8.	"ht 903".mp.
9.	melapure.mp.
10.	armonia.mp.
11.	melamil.mp.
12.	benedorm.mp.
13.	"bp 2013".mp.
14.	"BP2013".mp.
15.	"JL5DK93RCL".mp.
16.	1 or 2 or 3 or 4 or 5 or 6 or 7 or 8 or 9 or 10 or 11 or 12 or 13 or 14 or 15
17.	Delirium/
18.	Emergence Delirium/
19.	delirium.mp.
20.	"organic brain syndrome".mp.
21.	"acute confusion".mp.
22.	((postoperative or perioperative) adj1 delirium).mp.
23.	17 or 18 or 19 or 20 or 21 or 22
24.	16 and 23
25.	24
26.	limit 25 to yr="1990 -Current"
27.	26 use medall
28.	melatonin/
29.	melatonin*.mp.
30.	ramelteon.mp.
31.	rozerem.mp.
32.	"n-acetyl-5-methoxytryptamine".mp.
33.	melatoniin*.mp.
34.	circadian.mp.
35.	"ht 903".mp.
36.	melapure.mp.
37.	armonia.mp.
38.	melamil.mp.
39.	benedorm.mp.
40.	"bp 2013".mp.
41.	"BP2013".mp.
42.	"JL5DK93RCL".mp.
43.	28 or 29 or 30 or 31 or 32 or 33 or 34 or 35 or 36 or 37 or 38 or 39 or 40 or 41 or 42
44.	exp delirium/
45.	delirium.mp.
46.	"organic brain syndrome".mp.
47.	"acute confusion".mp.
48.	((postoperative or perioperative) adj1 delirium).mp.
49.	44 or 45 or 46 or 47 or 48
50.	43 and 49
51.	50
52.	limit 51 to yr="1990 -Current"
53.	52 use oemezd
54.	27 or 53
55.	54

Psychinfo: **Search Strategy**

Set#	Searched for
S1	MAINSUBJECT.EXACT("Melatonin")
S2	melatonin*
S3	ramelteon
S4	rozerem
S5	"n-acetyl-5-methoxytryptamine"
S6	melatoniin*
S7	circadian
S8	"ht 903"
S9	melapure
S10	armonia
S11	melamil
S12	benedorm
S13	"bp 2013"
S14	"bp2013"
S15	"JL5DK93RCL"
S16	OR(1:15)
S17	MAINSUBJECT.EXACT.EXPLODE("Delirium")
S18	Delirium
S19	"organic brain syndrome"
S20	"acute confusion"
S21	((postoperative or perioperative) N/1 delirium)
S22	OR(17:21)
S23	16 AND 22
S24	23 (limited to 1990-current)

Trials Registry

CONDITION: DELERIUM
OTHER: MELATONIN OR RAMELTEON OR ROZEREM OR n-acetyl-5-methoxytryptamine OR HT 903 OR MELAPURE OR ARMONIA OR MELAMIL OR BENEDORM OR JL5DK93RCL
STUDY TYPE: ALL
STUDY RESULTS: ALL

Appendix 2: List of studies excluded after full text review.

Study	Primary Reason for exclusion
Lam K et al. Effectiveness of Melatonin for the Prevention of Intensive Care Unit Delirium. *Pharmacotherapy*. 2019.	Wrong population
Bellapart J et al. Effect of Exogenous Melatonin Administration in Critically Ill Patients on Delirium and Sleep: A Randomized Controlled Trial. *Critical Care Research and Practice.* 2020	Wrong population
Bouajram R et al. Efficacy of sleep medication administration and impact on delirium in critically ill patients. *Critical Care Medicine*. 2021	Wrong population
Gandolfi J et al. The Effects of Melatonin Supplementation on Sleep Quality and Assessment of the Serum Melatonin in ICU Patients: A Randomized Controlled Trial. *Critical Care Medicine.* 2020	Wrong population
Jung S et al. Effectiveness of melatonin for the prevention of icu delirium: could it be dose-dependent? *Critical Care Medicine*. 2021	Wrong study design
Villa C et al. Enteral versus intravenous approach for the sedation of critically ill patients: A randomized and controlled trial. *Critical Care.* 2019	Wrong population
Daniels L at al. Pharmacologic Treatment of Intensive Care Unit Delirium and the Impact on Duration of Delirium, Length of Intensive Care Unit Stay, Length of Hospitalization, and 28-Day Mortality. *Mayo Clinic Proceedings.* 2018	Wrong population
Romero N et al. Evaluation of Delirium in Critically Ill Patients Prescribed Melatonin or Ramelteon. *Annals of Pharmacotherapy.* 2021	Wrong population
Romero N at al. Evaluation of melatonin and ramelteon for delirium prevention in critically ill patients. *Critical Care Medicine.* 2019.	Wrong population
Hamidi A et al. Documented sleep quality and scheduled sleep aid medication response in critically ill adults. *Critical Care Medicine.* 2019	Wrong population
Baumgartner L. Melatonin for the prevention of intensive care unit (ICU) delirium. *Critical Care Medicine.* 2018.	Wrong study design
Kikuchi N et al. Assessment of the relationship between hypnotics and delirium using the japanese adverse drug event report (JADER) database. *Yaguka Zasshi.* 2018	Wrong population
Ryo M et al. Efficacy of Ramelteon for delirium after lung cancer surgery. *Interactive Cardiovascular and Thoracic Surgery*. 2017	Wrong study design
Abbasi S et al. Potential role of exogenous melatonin supplement in delirium prevention in critically ill patients: A double-blind randomized pilot study. *Iranian Journal of Pharaceutical Research*. 2018	Wrong population
Forte M et al. The use of melatonin to prevent delirium in the critically ill elderly. *American Journal of Respiratory and Critical Care* Preventive role of ramelteon and suvorexant for postoperative delirium after pharyngolaryngectomy with esophagectomy *Medicine.* 2019	Wrong population
Kawada T. Preventive role of ramelteon and suvorexant for postoperative delirium after pharyngolaryngectomy with esophagectomy. *Esophagus.* 2018	Wrong study design
Hatta K et al. Real-World Effectiveness of Ramelteon and Suvorexant for Delirium Prevention in 948 Patients With Delirium Risk Factors. *The Journal of Clinical Psychiatry.* 2019	Wrong population

Wibrow B et al. Statistical analysis plan for the Prophylactic Melatonin for Delirium in Intensive Care (ProMEDIC): a randomised controlled trial. *Trials.* 2021	Wrong study design
Bobb E et al. Evaluation of melatonin on delirium prevention in the intensive care unit. *Critical Care Medicine.* 2019	Wrong study design
Fosnight S et al. Melatonin effect on incident delirium in hospitalized patients. *Journal of the American Geriatrics Society.* 2018.	Wrong population
Jouin G et al. Delirium in elderly patients: Successful use of melatonin. *International Journal of Clinical Pharmacy*. 2017	Wrong study design
Shi Y. Effects of Melatonin on Postoperative Delirium After PCI in Elderly Patients: A Randomized, Single-Center, Double-Blind, Placebo-Controlled Trial. *The Heart Surgery Forum.* 2021	Wrong population
Arizumi F et al. Efficacy of intervention for prevention of postoperative delirium after spine surgery. *Spine Surgery and Related Research.* 2021	Wrong study design
Xue ZJ et al. Assessing Preventative Effect of Ramelteon on Postoperative Delirium in Older Patients: Methodology Is Important. *American Journal of Geriatric Psychiatry.* 2021.	Wrong study design
Mu S et al. Relationship among melatonin, postoperative delirium, and postoperative cognitive dysfunction. *Annals of Palliative Medicine.* 2021	Wrong study design
Hokuto D et al. Preventative effects of ramelteon against postoperative delirium after elective liver surgery. *PLoS ONE*. 2020	Wrong study design
Shinsaku H et al. A phase II study of ramelteon for the prevention of postoperative delirium in elderly patients undergoing gastrectomy. *Surgery Today.* 2020	Wrong study design
Lopez C et al. Ramelteon for decreasing delirium in surgical intensive care unit patients. *Clinical Medicine Insights.* 2020.	Wrong population
Tsubosa Y et al. Postoperative delirium after pharyngolaryngectomy with esophagectomy: a role for ramelteon and suvorexant. *Esophagus*. 2017	Wrong study design
Welch S et al. Factors associated with delirium in surgical intensive care unit patients treated with supplemental melatonin: A case-cohort study. *Clinical neuropharmacology.* 2019	Wrong study design

Appendix 3: Risk of Bias Assessment

Risk of Bias (ROB) assessment

Trial	Ra.	As.	Ad.	Mi.	Me.	S.	Overall ROB
De Jonghe 2014	+	+	+	+	+	+	+
Ford 2020	+	+	+	+	+	+	+
Hashib *unpublished*	+	+	+	+	+	!	!
Gupta 2019	+	+	+	+	+	+	+
Jaiswal 2019	+	+	+	+	+	+	+
Mahrose 2021	+	!	!	+	!	+	!
Nickholgh 2011	+	+	+	+	+	+	+
Oh 2020	+	+	+	+	!	+	!
Sultan 2010	+	+	+	+	!	!	!
Yamaguchi 2014	+	!	+	+	+	+	!
Zadeh 2021	+	+	+	+	+	+	+

+ Low
! Some concern
- High

Ra. Randomisation process
As. Assignment to intervention
Ad. Adherence to intervention
Mi. Missing outcome data
Me. Measurement of outcome
S. Selection of results

Appendix 4: Funnel plot

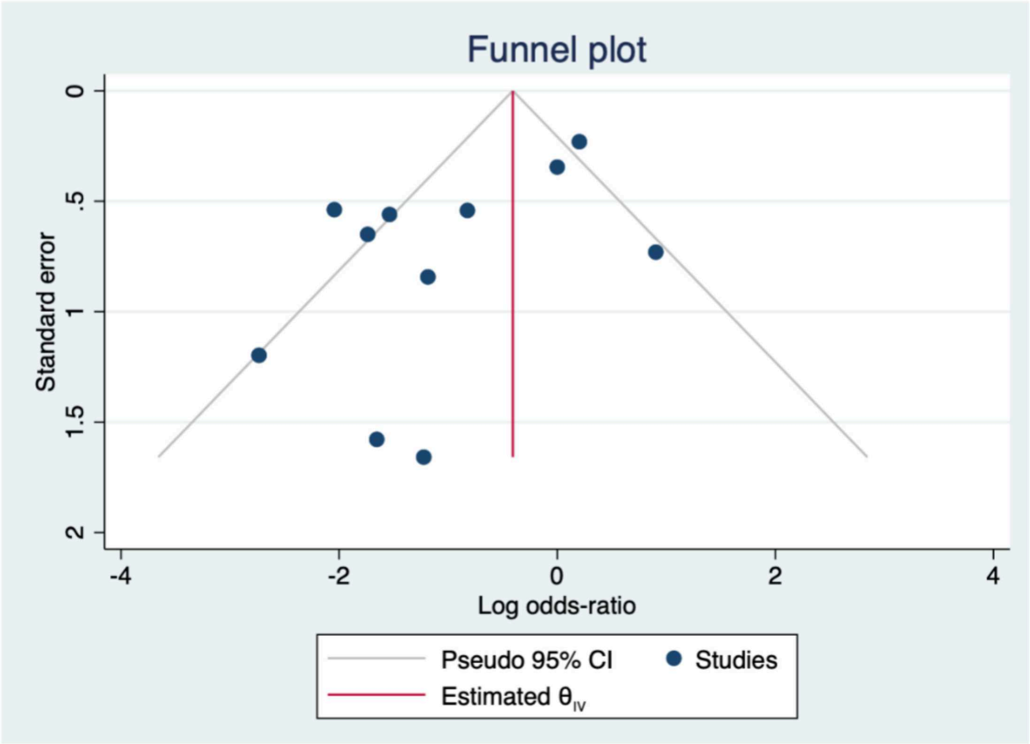

Supplement: Supplementary data [file bmjopen-2022-069950supp001.pdf]
