# Supplementary material for: Does melatonin administration reduce the incidence of postoperative delirium in adults? Systematic review and meta-analysis
Source: BMJ Open. 2023 Mar 29;13(3):e069950. doi: 10.1136/bmjopen-2022-069950 (PMC10069576; doi:10.1136/bmjopen-2022-069950)
Supplement: Author's manuscript [file bmjopen-2022-069950.draft_revisions.pdf]

BMJ Open

BMJ Open is committed to open peer review. As part of this commitment we make the peer review history of every article we publish publicly available.

When an article is published we post the peer reviewers' comments and the authors' responses online. We also post the versions of the paper that were used during peer review. These are the versions that the peer review comments apply to.

The versions of the paper that follow are the versions that were submitted during the peer review process. They are not the versions of record or the final published versions. They should not be cited or distributed as the published version of this manuscript.

BMJ Open is an open access journal and the full, final, typeset and author-corrected version of record of the manuscript is available on our site with no access controls, subscription charges or pay-per-view fees (http://bmjopen.bmj.com).

If you have any questions on BMJ Open's open peer review process please email info.bmjopen@bmj.com

BMJ Open

Melatonin for the prevention of postoperative delirium in adults: Systematic Review and Meta-analysis

Journal:	*BMJ Open*
Manuscript ID	bmjopen-2022-069950
Article Type:	Original research
Date Submitted by the Author:	07-Nov-2022
Complete List of Authors:	Barnes, Jonathan; Bristol Royal Infirmary<br>Sewart, Emma; North Bristol NHS Trust<br>Armstrong, Richard; University of Bristol<br>Pufulete, Maria; University of Bristol<br>Hinchliffe, Robert; University of Bristol, Population Health Sciences<br>Gibbison, Ben; Bristol Royal Infirmary<br>Mouton, Ronelle; Bristol Centre for Surgical Research, Dept population health sciences; North Bristol NHS Trust, Anaesthesia
Keywords:	Adult anaesthesia < ANAESTHETICS, CLINICAL PHARMACOLOGY, PSYCHIATRY, NEUROLOGY, THERAPEUTICS, SURGERY

SCHOLARONE™
Manuscripts

I, the Submitting Author has the right to grant and does grant on behalf of all authors of the Work (as defined in the below author licence), an exclusive licence and/or a non-exclusive licence for contributions from authors who are: i) UK Crown employees; ii) where BMJ has agreed a CC-BY licence shall apply, and/or iii) in accordance with the terms applicable for US Federal Government officers or employees acting as part of their official duties; on a worldwide, perpetual, irrevocable, royalty-free basis to BMJ Publishing Group Ltd ("BMJ") its licensees and where the relevant Journal is co-owned by BMJ to the co-owners of the Journal, to publish the Work in this journal and any other BMJ products and to exploit all rights, as set out in our licence.

The Submitting Author accepts and understands that any supply made under these terms is made by BMJ to the Submitting Author unless you are acting as an employee on behalf of your employer or a postgraduate student of an affiliated institution which is paying any applicable article publishing charge ("APC") for Open Access articles. Where the Submitting Author wishes to make the Work available on an Open Access basis (and intends to pay the relevant APC), the terms of reuse of such Open Access shall be governed by a Creative Commons licence – details of these licences and which Creative Commons licence will apply to this Work are set out in our licence referred to above.

Other than as permitted in any relevant BMJ Author's Self Archiving Policies, I confirm this Work has not been accepted for publication elsewhere, is not being considered for publication elsewhere and does not duplicate material already published. I confirm all authors consent to publication of this Work and authorise the granting of this licence.

For peer review only

Melatonin for the prevention of postoperative delirium in adults: Systematic Review and Meta-analysis

J. Barnes[1], E. Sewart[2], R. Armstrong[3], M. Pufulete[4], R. Hinchliffe[5], B. Gibbison[6], R. Mouton[7]

1 Specialty Trainee Anaesthesia and Intensive Care Medicine, Bristol Royal Infirmary, Bristol, UK
2 NIHR Academic Clinical Fellow in Anaesthesia, North Bristol Hospitals, Bristol, UK
3 NIHR Academic Clinical Fellow in Anaesthesia, University of Bristol, Bristol, UK
4 Senior Research Fellow, University of Bristol, Bristol, UK
5 Professor of Vascular Surgery, North Bristol Hospitals, Bristol, UK
6 Associate Professor of Cardiac Anaesthesia and Intensive Care, Bristol Royal Infirmary, Bristol, UK
7 Honorary Associate Professor and Consultant Anaesthetist, North Bristol Hospitals, Bristol, UK

Correspondence to

Jonathan Barnes
Jonathan.barnes4@nhs.net
University Hospitals Bristol and Weston
Bristol Royal Infirmary
Upper Maudlin Street
BS2 8HW

For peer review only

Structured Abstract

Postoperative delirium (POD) is a frequent problem after surgery, associated with increased morbidity and mortality. Many cases may be preventable and there is interest in the use of melatonin as a preventative agent.

Objective: This systematic review aims to provide an up-to-date synthesis of the available evidence on the effect of melatonin in the prevention of POD.

Design: A systematic search of randomised controlled trials of melatonin in POD was run across multiple databases (EMBASE, Medline, CINAHL, Psychinfo) and a clinical trials registry (clinicaltrials.org). Randomised controlled trials examining the effects of melatonin or melatonin receptor agonists and reporting outcomes related to POD are included.

Outcome measures: The primary outcome is incidence of POD. Secondary outcomes are duration of POD and length of hospital stay. Data synthesis was undertaken using a random-effects meta-analysis.

Results: Eleven studies, with 1244 patients from a range of surgical specialties were identified for inclusion. Seven studies used melatonin, in variable dosing schedules, and four used Ramelteon. The studies used eight different diagnostic tools to diagnose POD. Time points for assessment were also variable. Six studies were assessed as low risk of bias and five as some concern. The combined odds ratio of developing POD in the melatonin groups versus control was 0.41 (95% confidence interval, 0.21-0.80, p=0.01).

Conclusion: This review found melatonin may reduce the incidence of POD in adults undergoing surgery. However, included studies are heterogenous in their methodology. Further work to determine the optimum regime for melatonin administration, along with consensus of how best to evaluate results, would be beneficial.

Registration: This review was prospectively registered with PROSPERO (CRD42021285019).

Word count = 265

For peer review only

Strengths and Limitations

- This review provides an up-to-date assessment of the efficacy of melatonin for prevention of postoperative delirium.
- The search was performed across a range of databases and a clinical trials registry to ensure thorough data capture.
- Studies presented in this review include a diverse range of patients, surgical subspecialties and melatonin preparations and administration regimes.
- Results of this review are limited by the heterogeneity of the included studies limits, and lack of consistency of intervention design and outcome measures.
- Language restrictions to English may have led to exclusion of additional studies.

Protocol

Published protocol has been attached a separate file. *Sewart E, Barnes J, Armstrong RA, et al. Melatonin for the prevention of postoperative delirium in older adults: a protocol for a systematic review and meta-analysis. BMJ Open 2022;12:e063405. doi:10.1136/ bmjopen-2022-063405*

Funding Statement

This work was supported by the NIHR Biomedical Research Centre at University Hospitals Bristol and Weston NHS Foundation Trust and the University of Bristol. RA and ES are NIHR Academic Clinical Fellows. The views expressed in this publication are those of the author(s) and not necessarily those of the NHS, the National Institute for Health Research, the Department of Health and Social Care.

Competing Interests

None declared.

For peer review only - http://bmjopen.bmj.com/site/about/guidelines.xhtml

Introduction

Post-operative delirium (POD) is a common postoperative complication, especially in older patients [1,2]. POD comprises an acute and fluctuating disturbance in attention, awareness and cognition not explained by another neurocognitive disorder [3]. It usually occurs between one to three days after surgery [3]. Although there is no single universally accepted definition. POD is associated with a number of risk factors, including pain, bleeding, polypharmacy, sedative drugs and major surgery [4–8]. POD carries a significant healthcare and economic burden and is associated with an increased length of hospital stay, increased incidence of ongoing cognitive impairment, increased care needs and increased mortality [9–11]. It is thought that around 30-40% of cases may be preventable [12,13] and strategies to prevent POD have been highlighted as an important priority for healthcare systems [14].

Melatonin, a hormone produced by the pineal gland, is known to play an important role in circadian rhythm regulation [15] and is an attractive option as an agent for the prevention of POD. It has a low-risk side-effect profile and is inexpensive. Melatonin, and melatonin receptor agonists, are used therapeutically in the treatment of sleep-wake disorders [16] and jet lag [17] and may have a role in sedation, analgesia and as an anaesthetic adjunct [18]. Furthermore, melatonin has been identified as having a potential therapeutic role in several disorders of the mind including schizophrenia, depression and POD [19–21].

A previous systematic review of six studies (conducted in 2017) examining the use of melatonin and its agonists in the prevention of POD in older adults found some evidence to suggest a beneficial effect [22]. However, these studies were mostly small, heterogeneous in their methodology, and demonstrated conflicting results. Since then, multiple studies investigating melatonin and POD have been published.

The aim of this review is to provide an up to date assessment of melatonin as an intervention for the prevention of POD in adult patients. This review will also provide a description of how melatonin has been administered (dose, duration etc.) and how POD has been studied in randomised controlled trials (RCTs).

Methods

This study was developed using the Preferred Reporting Items for Systematic Reviews and Meta-Analyses (PRISMA) guidelines and checklist [23]. We prospectively registered the review PROSPERO (CRD42021285019) and the protocol has been published [24].

A search was conducted across EMBASE, Medline, CINAHL and Psychinfo for RCTs examining the effects of melatonin, or melatonin receptor agonists, in the prevention of POD. Only studies in adult patients (age 18 years or older) undergoing surgery were included. No restrictions were placed on type of surgery, the programme of drug administration (including timing, dose and additional treatments used) or on the intervention received by the comparator group. We included studies published in English between 1st January 1990 and 5th April 2022. This window was chosen in line with previous work [22], as it coincides with the development of the Beers Criteria, which identified medications that may cause or worsen delirium [25]. No limits were placed on the country of study. We also hand searched the reference lists of included studies. The search strategy is detailed in Appendix 1. The search was repeated using a clinical trials registry (clinicaltrials.org) to capture ongoing, but unpublished, studies. For any studies identified, and not yet published, the study lead was contacted to request results to be shared.

For peer review only - http://bmjopen.bmj.com/site/about/guidelines.xhtml

Citation management and data collection was undertaken using Covidence (Covidence, Melbourne, Australia) [26]. Titles and abstracts were independently screened by two reviewers (JB,RM). Full texts were then independently screened by the same two reviewers. All data was extracted in whole by two independent reviewers (JB, RM) using a pre-defined template. Discrepancies or disagreements at all stages were resolved by discussion between the two main reviewers. If there was still no consensus a third reviewer made the final decision (BG).

The key information gathered in the data collection form included:

1. Publication details: authors, country of study and years of study conduct and publication.
2. Participant demographics: sex, age, number, inclusion/exclusion criteria and surgery type.
3. Intervention details: drugs used, dose, timings, details of control interventions
4. Criteria and scales used for diagnosing and grading POD

The primary outcome of this review is the incidence of POD in adults undergoing surgery. The secondary outcomes are the duration of POD and length of postoperative hospital stay.

We used the Cochrane risk of bias tool, version 2, to assess risk of bias in five domains [27]: The randomisation process; deviations from the intended interventions (effects of assignment to intervention and effect of adhering to intervention); missing outcome data; measurement of the outcome and selection of the reported results. The trial quality and overall risk of bias were judged as low risk, high risk or some concerns. We inspected a funnel plot and used Egger's linear regression test to detect small study effects.

A qualitative synthesis and narrative description of the included studies are presented alongside meta-analysis of the primary outcome. All data analysis was undertaken using Stata (StataCorp LLC, Texas, USA) [28]. Categorical data are summarised by counts and percentages, and continuous data by means and standard deviations, unless otherwise indicated. Incidence of POD is summarised using odds ratios (with associated 95% confidence intervals) for individual studies and combined using random effect meta-analysis. Between-study heterogeneity is assessed using the I^2 statistic and random-effects estimates and a fixed-effects meta-analysis is also performed as a sensitivity analysis.

Results

The search identified 643 studies with 443 remaining after deduplication. A further 405 articles were excluded during screening of titles and abstracts. Of the 38 full-text reports assessed for eligibility, 14 were excluded due to the patient group and 14 were excluded for being the wrong study type. From the clinical trials search 46 studies were identified, 2 were duplicated in the literature search and were excluded. Six of the remaining studies met inclusion criteria and all study leads were contacted. One study was complete but unpublished and the author shared results via personal correspondence [29]. One author did not respond, and four studies were not in a position to share results (either not started recruiting or not completed data collection). This process is outlined in a PRISMA flowchart (Figure 1).

Figure 1: PRISMA flow chart for study selection process. N = number of articles

Eleven studies including 1244 participants were eligible for inclusion in this review. Characteristics of these studies and basic participant demographics are outlined in table 1. The studies included patients across a range of surgical specialities, including general, orthopaedic and cardiothoracic

For peer review only - http://bmjopen.bmj.com/site/about/guidelines.xhtml

surgery. One study included patients from a variety of surgical specialities [30]. Mean patient age was above 50 for all studies and several studies used a minimum age as an exclusion criterion.

For peer review only

Table 1: Characteristics of the studies and study participants

Author (year); Country	Year of study conduct	Drug (dose)(all once daily)		Timing of dosing	Duration of Therapy (days)	Study design / (blinding)	Reason for surgery	Patients N		Male patients N (%)		Age (years) Mean (SD) unless otherwise indicated		Assessment of delirium		Mean (SD) duration of surgery (minutes)	
		I	C					I	C	I	C	I	C	Scale	Timepoint	I	C
de Jonghe (2014); The Netherlands	2008-2012	Melatonin (3mg)	Placebo	Postoperative	5	RCT (double)	Hip fracture	186	192	53 (29)	62 (32)	84 (8)	83(8)	DSM-IV	Daily for 8 days post op (or until discharge)	NR	NR
Ford (2020); Australia	NR	Melatonin (3mg)	Placebo	Postoperative	7	RCT (double)	Cardiac	105	105	79 (75)	85 (81)	69 (8)	68 (8)	CAM or CAM-ICU (screening); DSM-V (scale)	Daily for 7 days if CAM positive	NR	NR
Gupta (2019); Indian	NR	Ramelteon (8mg)	Placebo	Preoperative	12 hours	RCT (double)	Mixed (requiring neuraxial anaesthesia)	50	50	35 (70)	33 (66)	69 (4)	71 (4)	CAM	Daily for 3 days	NR	NR
Hashim (unpublished); Iraq	2020	Melatonin (5mg)	No treatment	Preoperative	2 doses (night before & 90 mins pre op)	RCT (double)	NR	12	12	NR	NR	> 60	> 60	MDAS	30-, 60- and 90-minutes post-op	NR	NR
Jaiswal (2019); United States	2016-2017	Ramelteon (8mg)	Placebo	Postoperative	1 to 7	RCT (double)	Elective pulmonary thromboendarterectomy	59	28	29 (49)	29 (50)	58 (14)	56 (16)	CAM-ICU and RASS	Twice daily for 9 days (or until ICU discharge)	526 (480-540) ÷	510 (480-540)÷
Mahrose (2021); Egypt	NR	Melatonin (5mg) + Dexmedetomidine	Dexmedetomidine (bolus + infusion)	Preoperative/ postoperative	4	RCT (NR)	Elective coronary artery bypass graft	55	55	42 (76)	41 (75)	67 (7)	66 (6)	CAM or CAM-ICU	Twice daily for 5 days (or until discharge)	NR	NR
Nickkholgh (2011); Germany	2007-2009	Melatonin (50mg/kg)	Placebo	Intraoperative	1	RCT (double)	Liver resection	25	23	17 (68)	11 (48)	59 (10)	56 (11)	NR	Up to 7 days (frequency NR)	202 (80)	212 (79)
Oh (2020); United States	2017-2019	Ramelteon (8mg)	Placebo	Postoperative	3	RCT (quadruple)	Elective primary or revision hip or knee replacemen	41	39	14 (42)	14 (37)	74 (6)	75 (5)	DSM-V	Daily for 3 days	138 (72)	162 (84)

For peer review only

							t										
Sultan (2010); Egypt	NR	Melatonin (5mg)	No treatment	Postoperative	2	RCT (double)	Hip arthroplasty	53	49	24 (45)	22 (45)	70 (7)	72 (6)	AMT	Daily for 3 days	127 (45)	120 (37)
Yamaguchi ^ (2014); Japan	NR	Ramelteon (8mg)	Placebo	Postoperative	4	RCT (double)	Total knee arthroplasty	22	23	NR	NR	>=70	>=70	iCDSC	Thrice daily for 4 days	NR	NR
Zadeh (2021); Iran	2018-2019	Melatonin (3mg) prolonged release	Placebo		4	RCT (double)	Coronary artery bypass graft	30	30	20 (67)	22 (73)	60 (10)	63 (8)	CAM-ICU and MDAS	Twice daily for 48 hours post extubation	NR	NR

Table 1 footnotes:
^ Abstract only
NR – not reported
I – intervention group
C – control group
mg – milligrams
kg – kilograms
RCT – randomised control trial
SD – standard deviation
CAM – Confusion Assessment Method
CAM-ICU – Confusion Assessment Method for the Intensive Care Unit
DSM – Diagnostic and statistical manual of mental disorders
ICDSC – Intensive care delirium screening checklist score
AMT – Abbreviated Mental Test
MDAS.- Memorial Delirium Assessment Scale
*Median (range)
† Median (IQR)

For peer review only

Seven studies used melatonin as the study drug, in a variety of doses (from 3mg to 50mg/kg and in one study as a long acting formulation), with one using Dexmedetomidine alongside melatonin in the intervention arm [31]. Four studies used Ramelteon (always 8mg once daily). Dosing schedule was highly variable. Three studies included preoperative dosing (two exclusively preoperative, one pre and postoperative), in one study medication was given intraoperatively, whilst the others involved exclusively postoperative dosing. In studies with *a priori* dosing regimens, dosing programmes ranged from one to seven days. In two studies patients were included if they had had at least one or three doses respectively and in two other studies the duration of treatment was dependent on the length of hospital stay. Eight different measures of delirium were used across the identified studies. One study did not report the assessment tool used [32], however all other included studies used validated assessment tools. Some studies used multiple tools for diagnosis of delirium and assessing its severity and some tools were used in multiple ways (e.g. Confusion Assessment Measure was used a screening tool in one study and a diagnostic tool in others, whilst the Memorial Delirium Assessment Scale was used a diagnostic tool in one study and a tool to grade severity in another). The time points of assessment were also highly variable (table 1).

Incidence of delirium ranged from 0% to 42% in the intervention groups and 4% to 92% in the control groups. Pooled data showed evidence of a reduced incidence of POD associated with melatonin use (OR 0.41, 95% CI 0.21 to 0.80, 11 studies, 1244 participants, p=0.01). Assuming an overall risk of POD the same as that of the combined study populations (0.23), this gives a number needed to treat of 8.40. Random-effects estimates were used as there is significant between-study heterogeneity (I^2 =71.97%) (Figure 2). However, sensitivity analysis with a fixed-effects meta-analysis gave similar results, also finding a reduced incidence of POD associated with melatonin use (OR 0.63, 95% CI 0.48-0.83, p<0.01).

Figure 2: Forest plot displaying odds ratios and 95% confidence intervals of effect of Melatonin or melatonergic agent on incidence of POD per study. Overall odds ratio generated using a random effects meta analysis.

Duration of POD and length of hospital stay were each reported by five studies (Table 2). The median durations of delirium reported by ranged from 0.7 days [33] to 3 days [34]. Mean duration of hospital stay ranged from 7 to 17 days. Due to the paucity of reported data, neither synthesis of secondary outcome data nor reporting of complications associated with treatment were performed.

For peer review only

Table 2: Study outcomes

Author (year); Country	Delirium incidence N (%)		Delirium duration (days) median (range)		Hospital stay (days) Mean (SD) unless otherwise indicated	
	**I**	**C**	**I**	**C**	**I**	**C**
de Jonghe (2014); The Netherlands	55 (30)	49 (25.5)	2 (1-3)	2 (1-3)	11 (6-14.5)*	11 (8-17)*
Ford (2020); Australia	21 (21)	21 (20)	3 (2-4)†	2 (1-3)†	8 (6-10)†	7 (6-8)†
Gupta (2019); Indian	2 (4)	6 (12)	NR	NR	NR	NR
Hashim (unpublished); Iraq	5 (42)	11 (92)	NR	NR	NR	NR
Jaiswal (2019); United States	19 (32)	22 (38)	1.0 (0.6-1.5)†	0.7 (0.4-1.2)†	12 (10-16)†	12 (10-14)†
Mahrose (2021); Egypt	6 (11)	12 (27)	1.0 (+- 0.3)	2.0 (+- 0.6)	12 (+-5)	14 (+-6)
Nickkholgh (2011); Germany	0	1 (4)	NR	NR	13.5 (+-1.5)	17 (+-2)
Oh (2020); United States	7 (21)**	3 (8)**	1.3 (+-0.5)	1.2 (+-0.5)	NR	NR
Sultan (2010); Egypt	5 (9)	16 (33)	NR	NR	NR	NR
Yamaguchi (2014); Japan	0	2 (9)	NR	NR	NR	NR
Zadeh (2021); Iran	4 (13)**	14 (47)**	NR	NR	NR	NR

I – Intervention group

For peer review only - http://bmjopen.bmj.com/site/about/guidelines.xhtml

C - Control group
NR – Not reported
SD – Standard deviation
* Median (Range)
⸸ Median (IQR)
** delirium incidence reported at more than one timepoint. The highest reported rate is included here.

For peer review only

All included studies used some blinding and randomisation process and a validated outcome tool(s). Six studies were considered at low risk of bias, five were rated as "some concern." This occurred due to studies lacking either a clearly pre-specified analysis plan and/or concerns regarding the blinding process. No studies were considered at high risk of bias (Appendix 2). A funnel plot was prepared and inspected, with input from an expert statistician (Appendix 3). This displayed no clear evidence of reporting bias.

Certainty of evidence was assessed using the Grading of Recommendations Assessment, Development and Evaluation (GRADE) approach. Results are outlined in Table 3. Overall, evidence included in this review supports melatonin as effective prophylaxis for delirium with a moderate level of certainty. Recommendation was rated down due to heterogeneity/inconsistency, which could not be explained as subgroup analysis was unable to be undertaken.

Table 3: GRADE assessment of evidence certainty, Melatonin compared to placebo for prevention of post-operative delirium

Melatonin compared to placebo for prevention of post-operative delirium

Patient or population: Adult patients undergoing surgery
Setting: Hospital
Intervention: Melatonin or melatonin receptor agonist
Comparison: Placebo

Outcomes	Anticipated absolute effects		Relative effect (95% CI)	№ of participants (studies)	Certainty of the evidence (GRADE)	Comments
	Risk with placebo	Risk with melatonin				
Delirium	Study population		OR 0.41 (0.21 to 0.80)	1244 (11 RCTs)	⊕⊕⊕ MODERATE	Low risk of bias, precise, no evidence of publication bias. Rated down for inconsistency. Delirium incidence was recorded at multiple timepoints, this refers to "any delirium" during study window.
	157/606	124/638				

*The risk in the intervention group (and its 95% confidence interval) is based on the assumed risk in the comparison group and the relative effect of the intervention (and its 95% CI).

CI: Confidence interval; RR: Risk ratio; OR: Odds ratio;

GRADE Working Group grades of evidence
High certainty: We are very confident that the true effect lies close to that of the estimate of the effect
Moderate certainty: We are moderately confident in the effect estimate: The true effect is likely to be close to the estimate of the effect, but there is a possibility that it is substantially different
Low certainty: Our confidence in the effect estimate is limited: The true effect may be substantially different from the estimate of the effect
Very low certainty: We have very little confidence in the effect estimate: The true effect is likely to be substantially different from the estimate of effect

Discussion

The main findings from this systematic review are that melatonin and melatonin receptor agonists are associated with a reduced incidence of POD in adult patients undergoing surgery, whilst huge variation exists in the intervention design as well as the outcome measures employed.

For peer review only

A recent RCT found no impact of melatonin on incidence of POD in adult intensive care patients [35]. However, this study assessed only critically unwell patients who started taking melatonin treatment only once they had been admitted to intensive care. This therefore does not rule out potential benefit of melatonin in different patient groups and in different treatment regimens. The results from our review concur with the findings from an earlier review on a similar postoperative patient population [22]. There has been further interest in the effects of melatonin on postoperative delirium since the publication of the review by Campbell et al., and we included six additional recently published, and one unpublished, RCTs.

Multiple databases were searched, along with hand searching of references and a registry search, ensuring good capture of includable studies. No included studies were at high risk of bias and those included captured wide variety of patient groups and surgical specialities. Alongside qualitative analysis of the available evidence this study also adds quantitative evaluation of synthesised data on the effect of melatonin on preventing POD. Our review is limited by the heterogeneity of the published trials of melatonin for the prevention of POD. The study interventions were highly variable, including populations, drugs used, dosing and administration regimens. The outcome measures used varied widely across studies, and there was little consistency in when assessments were undertaken, or how frequently. Given delirium has a fluctuant nature, this may have contributed to under-reporting of delirium in several trials. The lack of consistency of either intervention or outcome measure also makes it challenging to conclude what an optimal melatonin dosing schedule would look like, and evaluation of this would be a valuable area for further study. Most studies excluded patients with pre-existing cognitive impairment and those on known psychoactive medications. These patients represent some of the highest risk of POD and would therefore arguably be some of those most likely to benefit from POD reduction strategies. Furthermore, the interventions used in the included studies may not reflect the likely use of melatonin in practice, limiting the external validity of these results. It is likely that future POD prevention strategies will involve multi-faceted care bundles, rather than single interventions (e.g. non pharmaceutical sleep hygiene strategies, pro-active pain management, avoidance of other exacerbating medications etc. along with any pharmaceutical agent(s)).

In conclusion, melatonin has both biologic plausibility and clinical evidence to support it as a treatment to prevent POD. However, the optimum protocol for melatonin use is yet to be determined. A consensus on how best to deliver the intervention and evaluate results is needed before further, adequately-powered clinical evaluation can be performed to properly analyse melatonin as a preventative intervention for delirium. Clinical applicability of findings from future work may be enhanced by focusing on those at highest risk of POD.

Ethics and dissemination

No ethical approval is required for this manuscript. Findings will be presented through conferences and peer reviewed publication.

Author contributions

JB and ES are joint first authors and RM and BG are joint final authors.
All authors have contributed fully to the concept and design of the review for which this protocol has been written. JB, ES, BG and RM wrote and reviewed the manuscript before submission. RA supervised the meta-analysis. MP, RA and RJH reviewed and edited the manuscript before submission. RM is the guarantor of the review.

For peer review only

Acknowledgements

We thank Professor Jonathan Sterne, professor of medical statistics and epidemiology, for his assistance with assessment of potential reporting bias and Sarah Rudd, Knowledge Specialist, for her assistance with the literature search.

Word count: 3540 (incl. tables, excl. abstract)

For peer review only

References

1. Inouye SK. Delirium in older persons. *The New England Journal of Medicine* 2006; **354**: 1157–65.

2. Inouye SK, Westendorp RGJ, Saczynski JS. Delirium in elderly people. *Lancet (London, England)* 2014; **383**: 911–22.

3. American Psychiatric Association. *Diagnostic and Statistical Manual of Mental Disorders: DSM-5.*, 5th edition. Washington, D.C.: American Psychiatric Publishing., 2013.

4. Inouye SK, Charpentier PA. Precipitating factors for delirium in hospitalized elderly persons. Predictive model and interrelationship with baseline vulnerability. *JAMA* 1996; **275**: 852–7.

5. Vaurio LE, Sands LP, Wang Y, Mullen EA, Leung JM. Postoperative delirium: the importance of pain and pain management. *Anesthesia and Analgesia* 2006; **102**: 1267–73.

6. Oldroyd C, Scholz AFM, Hinchliffe RJ, McCarthy K, Hewitt J, Quinn TJ. A systematic review and meta-analysis of factors for delirium in vascular surgical patients. *Journal of Vascular Surgery* 2017; **66**: 1269-1279.e9.

7. Galyfos GC, Geropapas GE, Sianou A, Sigala F, Filis K. Risk factors for postoperative delirium in patients undergoing vascular surgery. *Journal of Vascular Surgery* 2017; **66**: 937–46.

8. Bilotta F, Lauretta MP, Borozdina A, Mizikov VM, Rosa G. Postoperative delirium: risk factors, diagnosis and perioperative care. *Minerva Anestesiologica* 2013; **79**: 1066–76.

9. Leslie DL, Zhang Y, Holford TR, Bogardus ST, Leo-Summers LS, Inouye SK. Premature death associated with delirium at 1-year follow-up. *Archives of Internal Medicine* 2005; **165**: 1657–62.

10. Buurman BM, Hoogerduijn JG, de Haan RJ et al. Geriatric conditions in acutely hospitalized older patients: prevalence and one-year survival and functional decline. *PloS One* 2011; **6**: e26951.

11. Pitkala KH, Laurila JV, Strandberg TE, Tilvis RS. Prognostic significance of delirium in frail older people. *Dementia and Geriatric Cognitive Disorders* 2005; **19**: 158–63.

12. Inouye SK, Bogardus ST, Charpentier PA et al. A multicomponent intervention to prevent delirium in hospitalized older patients. *The New England Journal of Medicine* 1999; **340**: 669–76.

13. Marcantonio ER, Flacker JM, Wright RJ, Resnick NM. Reducing delirium after hip fracture: a randomized trial. *Journal of the American Geriatrics Society* 2001; **49**: 516–22.

14. O'Mahony R, Murthy L, Akunne A, Young J. Synopsis of the National Institute for Health and Clinical Excellence Guideline for Prevention of Delirium. *Annals of Internal Medicine* 2011; **154**: 746–51.

15. Zisapel N. New perspectives on the role of melatonin in human sleep, circadian rhythms and their regulation. *British Journal of Pharmacology* 2018; **175**: 3190–9.

16. Buscemi N, Vandermeer B, Hooton N et al. Efficacy and safety of exogenous melatonin for secondary sleep disorders and sleep disorders accompanying sleep restriction: meta-analysis. *BMJ (Clinical research ed.)* 2006; **332**: 385–93.

17. Petrie K, Conaglen JV, Thompson L, Chamberlain K. Effect of melatonin on jet lag after long haul flights. *BMJ : British Medical Journal* 1989; **298**: 705–7.

For peer review only

18. Procaccini D, Lobner K, Azamfirei R, Kudchadkar SR. Melatonin for anaesthetic indications in paediatric patients: a systematic review. *Anaesthesia* 2021; **76**: 837–49.

19. Morera-Fumero AL, Abreu-Gonzalez P. Role of melatonin in schizophrenia. *International Journal of Molecular Sciences* 2013; **14**: 9037–50.

20. Cardinali DP, Srinivasan V, Brzezinski A, Brown GM. Melatonin and its analogs in insomnia and depression. *Journal of Pineal Research* 2012; **52**: 365–75.

21. Scholtens RM, de Rooij SEJA, Vellekoop AE, Vrouenraets BC, van Munster BC. Preoperative CSF Melatonin Concentrations and the Occurrence of Delirium in Older Hip Fracture Patients: A Preliminary Study. *PloS One* 2016; **11**: e0167621.

22. Campbell AM, Axon DR, Martin JR, Slack MK, Mollon L, Lee JK. Melatonin for the prevention of postoperative delirium in older adults: a systematic review and meta-analysis. *BMC Geriatrics* 2019; **19**: 272.

23. Page MJ, McKenzie JE, Bossuyt PM et al. The PRISMA 2020 statement: an updated guideline for reporting systematic reviews. *BMJ* 2021; **372**: n71.

24. Sewart E, Barnes J, Armstrong RA et al. Melatonin for the prevention of postoperative delirium in older adults: a protocol for a systematic review and meta-analysis. *BMJ Open* 2022; **12**: e063405.

25. Beers MH, Ouslander JG, Rollingher I, Reuben DB, Brooks J, Beck JC. Explicit criteria for determining inappropriate medication use in nursing home residents. UCLA Division of Geriatric Medicine. *Archives of Internal Medicine* 1991; **151**: 1825–32.

26. Veritas Health Innovation. Covidence systematic review software.https://www.covidence.org/.

27. Sterne JAC, Savović J, Page MJ et al. RoB 2: a revised tool for assessing risk of bias in randomised trials. *BMJ* 2019; **366**: l4898.

28. StataCorp. Stata Statistical Software: Release 17. 2021.

29. Hashim H. The efficacy of oral Melatonin in preventing Postoperative Delirium for patients undergoing orthopedic surgery under general anesthesia: A randomized controlled trial. *Unpublished*2022.

30. Gupta P, Verma R, Kohli M, Shukla N, Kannaujia S. The Effect of Ramelteon on Postoperative Delirium in Elderly Patients: A Randomised Double-Blind Study. *JOURNAL OF CLINICAL AND DIAGNOSTIC RESEARCH*2019; doi 10.7860/jcdr/2019/42635.13384.

31. Mahrose R, ElSerwi H, Maurice A, Elsersi M. Postoperative delirium after coronary artery bypass graft surgery: Dexmedetomidine infusion alone or with the addition of oral melatonin. *Egyptian Journal of Anaesthesia* 2021; **37**: 62–8.

32. Nickkholgh A, Schneider H, Sobirey M et al. The use of high-dose melatonin in liver resection is safe: first clinical experience. *Journal of Pineal Research* 2011; **50**: 381–8.

33. Jaiswal SJ, Vyas AD, Heisel AJ et al. Ramelteon for Prevention of Postoperative Delirium: A Randomized Controlled Trial in Patients Undergoing Elective Pulmonary Thromboendarterectomy. *Critical Care Medicine* 2019; **47**: 1751–8.

For peer review only

34. Ford AH, Flicker L, Kelly R et al. The Healthy Heart-Mind Trial: Randomized Controlled Trial of Melatonin for Prevention of Delirium. *Journal of the American Geriatrics Society* 2020; **68**: 112–9.

35. Wibrow B, Martinez FE, Myers E et al. Prophylactic melatonin for delirium in intensive care (Pro-MEDIC): a randomized controlled trial. *Intensive Care Medicine* 2022; **48**: 414–25.

For peer review only

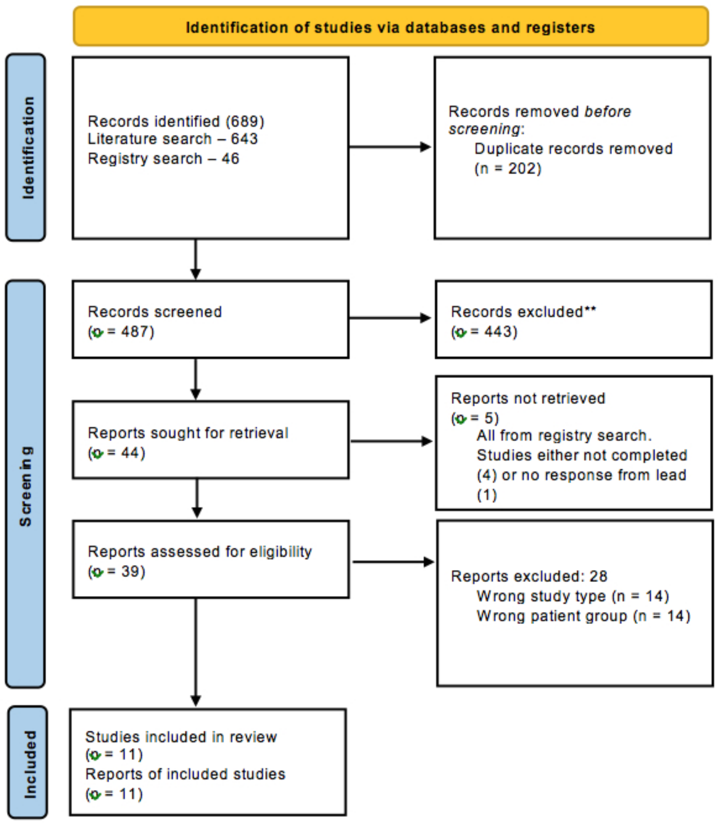

*Consider, if feasible to do so, reporting the number of records identified from each database or register searched (rather than the total number across all databases/registers).

**If automation tools were used, indicate how many records were excluded by a human and how many were excluded by automation tools.

Figure 1: PRISMA flow chart for study selection process. N = number of articles

360x382mm (57 x 57 DPI)

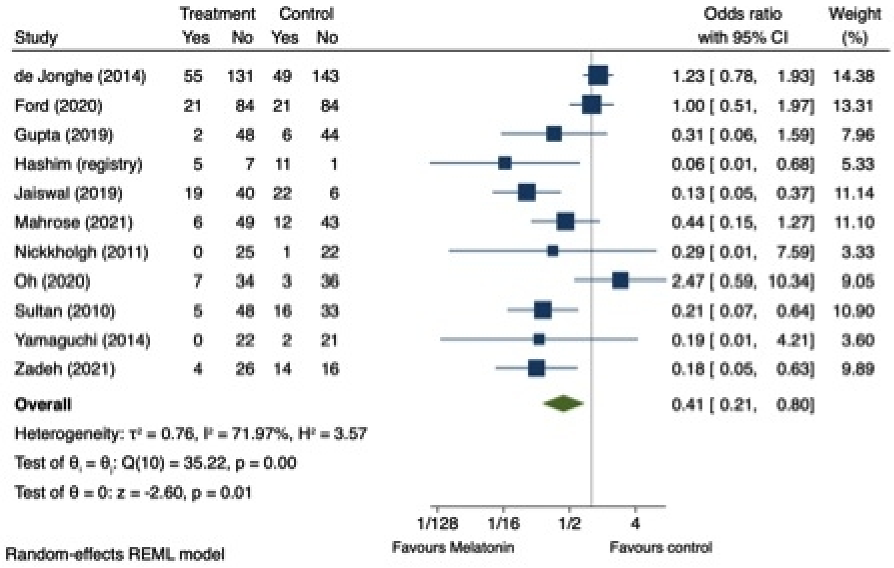

Figure 2: Forest plot displaying odds ratios and 95% confidence intervals of effect of Melatonin or melatonergic agent on incidence of POD per study. Overall odds ratio generated using a random effects meta analysis.

159x102mm (72 x 72 DPI)

Appendix 1: Search Strategy

[melatonin(MH) OR ramelteon(TW) OR rozerem(TW) OR "melatonin agonist*"(TW) OR "N-Acetyl-5-methoxytryptamine"(TW) OR melatonin*(TW) OR Melatoniin*(TW) OR circadin(TW) OR "HT 903"(TW) OR melapure(TW) OR armonia(TW) OR melamil(TW) OR benedorm(TW) OR "BP 2013"(TW) OR BP2013 OR JL5DK93RCL(TW)]

AND

[delirium(MH) OR "emergence delirium"(MH), OR delirium(TW) OR "perioperative delirium"(TW) OR "postoperative delirium"(TW) OR "organic brain syndrome"(TW) OR "acute confusion"(TW)]

Dates: 1st January 1990 – 5th April 2022

Other limits: Nil

For peer review only

Appendix 2: Risk of Bias Assessment

Risk of Bias (ROB) assessment

Trial	Ra.	As.	Ad.	Mi.	Me.	S.	Overall ROB
De Jonghe 2014	+	+	+	+	+	+	+
Ford 2020	+	+	+	+	+	+	+
Hashib *unpublished*	+	+	+	+	+	!	!
Gupta 2019	+	+	+	+	+	+	+
Jaiswal 2019	+	+	+	+	+	+	+
Mahrose 2021	+	!	!	+	!	+	!
Nickholgh 2011	+	+	+	+	+	+	+
Oh 2020	+	+	+	+	!	+	!
Sultan 2010	+	+	+	+	!	!	!
Yamaguchi 2014	+	!	+	+	+	+	!
Zadeh 2021	+	+	+	+	+	+	+

+ Low
! Some concern
- High

Ra. Randomisation process
As. Assignment to intervention
Ad. Adherence to intervention
Mi. Missing outcome data
Me. Measurement of outcome
S. Selection of results

view only

Appendix 3: Funnel plot

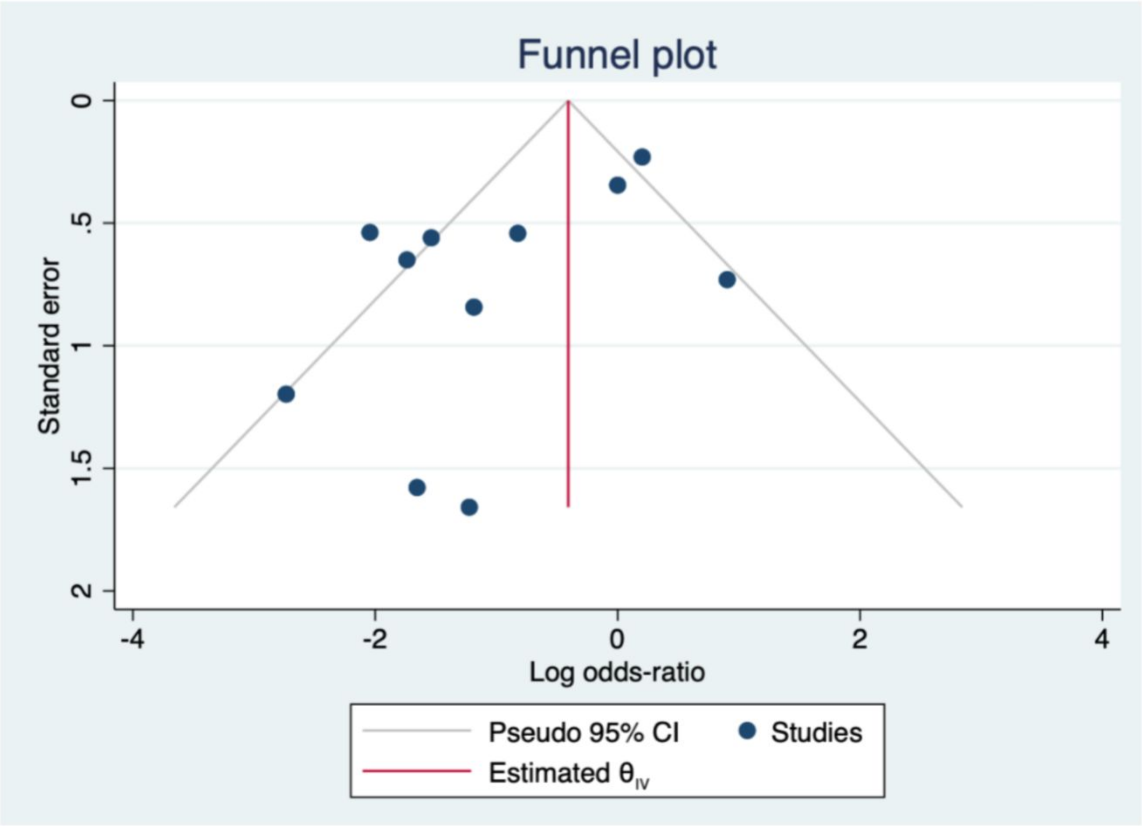

view only

PRISMA 2020 Checklist

Section and Topic	Item #	Checklist item	Location where item is reported
**TITLE**			
Title	1	Identify the report as a systematic review.	Page 1
**ABSTRACT**			
Abstract	2	See the PRISMA 2020 for Abstracts checklist.	Page 2
**INTRODUCTION**			
Rationale	3	Describe the rationale for the review in the context of existing knowledge.	Intro (p4)
Objectives	4	Provide an explicit statement of the objective(s) or question(s) the review addresses.	Intro (p4)
**METHODS**			
Eligibility criteria	5	Specify the inclusion and exclusion criteria for the review and how studies were grouped for the syntheses.	Methods (p4)
Information sources	6	Specify all databases, registers, websites, organisations, reference lists and other sources searched or consulted to identify studies. Specify the date when each source was last searched or consulted.	Methods (p4)
Search strategy	7	Present the full search strategies for all databases, registers and websites, including any filters and limits used.	Appendix 1
Selection process	8	Specify the methods used to decide whether a study met the inclusion criteria of the review, including how many reviewers screened each record and each report retrieved, whether they worked independently, and if applicable, details of automation tools used in the process.	Methods (p3/4)
Data collection process	9	Specify the methods used to collect data from reports, including how many reviewers collected data from each report, whether they worked independently, any processes for obtaining or confirming data from study investigators, and if applicable, details of automation tools used in the process.	Methods (p3/4)
Data items	10a	List and define all outcomes for which data were sought. Specify whether all results that were compatible with each outcome domain in each study were sought (e.g. for all measures, time points, analyses), and if not, the methods used to decide which results to collect.	Methods (p3/4)
	10b	List and define all other variables for which data were sought (e.g. participant and intervention characteristics, funding sources). Describe any assumptions made about any missing or unclear information.	Methods (p3/4)
Study risk of bias assessment	11	Specify the methods used to assess risk of bias in the included studies, including details of the tool(s) used, how many reviewers assessed each study and whether they worked independently, and if applicable, details of automation tools used in the process.	Cochrane ROB (results P12, appendix 2)
Effect measures	12	Specify for each outcome the effect measure(s) (e.g. risk ratio, mean difference) used in the synthesis or presentation of results.	Results P5-12
Synthesis methods	13a	Describe the processes used to decide which studies were eligible for each synthesis (e.g. tabulating the study intervention characteristics and comparing against the planned groups for each synthesis (item #5)).	Methods
	13b	Describe any methods required to prepare the data for presentation or synthesis, such as handling of missing summary statistics, or data conversions.	Methods
	13c	Describe any methods used to tabulate or visually display results of individual studies and syntheses.	Methods
	13d	Describe any methods used to synthesize results and provide a rationale for the choice(s). If meta-analysis was performed, describe the model(s), method(s) to identify the presence and extent of statistical heterogeneity, and software package(s) used.	Methods
	13e	Describe any methods used to explore possible causes of heterogeneity among study results (e.g. subgroup analysis, meta-regression).	Methods/appendix
	13f	Describe any sensitivity analyses conducted to assess robustness of the synthesized results.	N/a
Reporting bias assessment	14	Describe any methods used to assess risk of bias due to missing results in a synthesis (arising from reporting biases).	Methods/appendix
Certainty assessment	15	Describe any methods used to assess certainty (or confidence) in the body of evidence for an outcome.	Methods/results (GRADE)

For peer review only - http://bmjopen.bmj.com/site/about/guidelines.xhtml

PRISMA 2020 Checklist

Section and Topic	Item #	Checklist item	Location where item is reported
**RESULTS**			
Study selection	16a	Describe the results of the search and selection process, from the number of records identified in the search to the number of studies included in the review, ideally using a flow diagram.	Results
	16b	Cite studies that might appear to meet the inclusion criteria, but which were excluded, and explain why they were excluded.	N/a
Study characteristics	17	Cite each included study and present its characteristics.	Table 1/2
Risk of bias in studies	18	Present assessments of risk of bias for each included study.	Appendix 2
Results of individual studies	19	For all outcomes, present, for each study: (a) summary statistics for each group (where appropriate) and (b) an effect estimate and its precision (e.g. confidence/credible interval), ideally using structured tables or plots.	Results
Results of syntheses	20a	For each synthesis, briefly summarise the characteristics and risk of bias among contributing studies.	Results/appendix
	20b	Present results of all statistical syntheses conducted. If meta-analysis was done, present for each the summary estimate and its precision (e.g. confidence/credible interval) and measures of statistical heterogeneity. If comparing groups, describe the direction of the effect.	Results
	20c	Present results of all investigations of possible causes of heterogeneity among study results.	Results/appendix
	20d	Present results of all sensitivity analyses conducted to assess the robustness of the synthesized results.	N/a
Reporting biases	21	Present assessments of risk of bias due to missing results (arising from reporting biases) for each synthesis assessed.	Results/appendix
Certainty of evidence	22	Present assessments of certainty (or confidence) in the body of evidence for each outcome assessed.	Results
**DISCUSSION**			
Discussion	23a	Provide a general interpretation of the results in the context of other evidence.	Discussion
	23b	Discuss any limitations of the evidence included in the review.	Discussion
	23c	Discuss any limitations of the review processes used.	DIscussion
	23d	Discuss implications of the results for practice, policy, and future research.	Discussion
**OTHER INFORMATION**			
Registration and protocol	24a	Provide registration information for the review, including register name and registration number, or state that the review was not registered.	Abstract/Methods
	24b	Indicate where the review protocol can be accessed, or state that a protocol was not prepared.	Abstract/methods
	24c	Describe and explain any amendments to information provided at registration or in the protocol.	N/a
Support	25	Describe sources of financial or non-financial support for the review, and the role of the funders or sponsors in the review.	Funding Statement
Competing interests	26	Declare any competing interests of review authors.	Competing interests
Availability of data, code and other materials	27	Report which of the following are publicly available and where they can be found: template data collection forms; data extracted from included studies; data used for all analyses; analytic code; any other materials used in the review.	References/appendix

From: Page MJ, McKenzie JE, Bossuyt PM, Boutron I, Hoffmann TC, Mulrow CD, et al. The PRISMA 2020 statement: an updated guideline for reporting systematic reviews. BMJ 2021;372:n71. doi: 10.1136/bmj.n71

For peer review only - http://bmjopen.bmj.com/site/about/guidelines.xhtml

For more information, visit: http://www.prisma-statement.org/

For peer review only

BMJ Open

Does melatonin administration reduce the incidence of postoperative delirium in adults? Systematic Review and Meta-analysis

Journal:	*BMJ Open*
Manuscript ID	bmjopen-2022-069950.R1
Article Type:	Original research
Date Submitted by the Author:	01-Feb-2023
Complete List of Authors:	Barnes, Jonathan; Bristol Royal Infirmary, Anaesthesia<br>Sewart, Emma; North Bristol NHS Trust<br>Armstrong, Richard; University of Bristol<br>Pufulete, Maria; University of Bristol<br>Hinchliffe, Robert; University of Bristol, Population Health Sciences<br>Gibbison, Ben; Bristol Royal Infirmary<br>Mouton, Ronelle; Bristol Centre for Surgical Research, Dept population health sciences; North Bristol NHS Trust, Anaesthesia
**Primary Subject Heading**:	Anaesthesia
Secondary Subject Heading:	Anaesthesia, Neurology, Mental health, Pharmacology and therapeutics
Keywords:	Adult anaesthesia < ANAESTHETICS, CLINICAL PHARMACOLOGY, PSYCHIATRY, NEUROLOGY, THERAPEUTICS, SURGERY

SCHOLARONE™
Manuscripts

I, the Submitting Author has the right to grant and does grant on behalf of all authors of the Work (as defined in the below author licence), an exclusive licence and/or a non-exclusive licence for contributions from authors who are: i) UK Crown employees; ii) where BMJ has agreed a CC-BY licence shall apply, and/or iii) in accordance with the terms applicable for US Federal Government officers or employees acting as part of their official duties; on a worldwide, perpetual, irrevocable, royalty-free basis to BMJ Publishing Group Ltd ("BMJ") its licensees and where the relevant Journal is co-owned by BMJ to the co-owners of the Journal, to publish the Work in this journal and any other BMJ products and to exploit all rights, as set out in our licence.

The Submitting Author accepts and understands that any supply made under these terms is made by BMJ to the Submitting Author unless you are acting as an employee on behalf of your employer or a postgraduate student of an affiliated institution which is paying any applicable article publishing charge ("APC") for Open Access articles. Where the Submitting Author wishes to make the Work available on an Open Access basis (and intends to pay the relevant APC), the terms of reuse of such Open Access shall be governed by a Creative Commons licence – details of these licences and which Creative Commons licence will apply to this Work are set out in our licence referred to above.

Other than as permitted in any relevant BMJ Author's Self Archiving Policies, I confirm this Work has not been accepted for publication elsewhere, is not being considered for publication elsewhere and does not duplicate material already published. I confirm all authors consent to publication of this Work and authorise the granting of this licence.

For peer review only

Does melatonin administration reduce the incidence of postoperative delirium in adults? Systematic Review and Meta-analysis

J. Barnes[1], E. Sewart[2], R. Armstrong[3], M. Pufulete[4], R. Hinchliffe[5], B. Gibbison[6], R. Mouton[7]

1 Specialty Trainee Anaesthesia and Intensive Care Medicine, Bristol Royal Infirmary, Bristol, UK
2 NIHR Academic Clinical Fellow in Anaesthesia, North Bristol Hospitals, Bristol, UK
3 NIHR Academic Clinical Fellow in Anaesthesia, University of Bristol, Bristol, UK
4 Senior Research Fellow, University of Bristol, Bristol, UK
5 Professor of Vascular Surgery, North Bristol Hospitals, Bristol, UK
6 Associate Professor of Cardiac Anaesthesia and Intensive Care, Bristol Royal Infirmary, Bristol, UK
7 Honorary Associate Professor and Consultant Anaesthetist, North Bristol Hospitals, Bristol, UK

Correspondence to
Jonathan Barnes
Jonathan.barnes4@nhs.net
University Hospitals Bristol and Weston
Bristol Royal Infirmary
Upper Maudlin Street
BS2 8HW

For peer review only

Structured Abstract

Postoperative delirium (POD) is common. It is associated with increased morbidity and mortality. Many cases may be preventable and melatonin offers promise as a preventative agent.

Objective: This systematic review provides an up-to-date synthesis of the evidence on the effect of melatonin in preventing POD.

Design: A systematic search of randomised controlled trials of melatonin in POD was run across multiple databases (EMBASE, Medline, CINAHL, Psychinfo) and a clinical trials registry (clinicaltrials.org) (1/1/1990 – 5/4/2022). Studies examining the effects of melatonin on POD incidence in adults are included. Risk of bias was assessed using the Cochrane risk of bias 2 tool.

Outcome measures: The primary outcome is POD incidence. Secondary outcomes are POD duration and length of hospital stay. Data synthesis was undertaken using a random-effects meta-analysis and presented using forest plots. A summary of methodology and outcome measures in included studies is also presented.

Results: Eleven studies, with 1244 patients from a range of surgical specialties were included. Seven studies used melatonin, in variable doses, and four used Ramelteon. Eight different diagnostic tools were used to diagnose POD. Time points for assessment also varied. Six studies were assessed as low risk of bias and five as some concern. The combined odds ratio of developing POD in the melatonin groups versus control was 0.41 (95% confidence interval, 0.21-0.80, p=0.01).

Conclusion: This review found melatonin may reduce the incidence of POD in adults undergoing surgery. However, included studies displayed inconsistency in their methodology and outcome reporting. Further work to determine the optimum regime for melatonin administration, along with consensus of how best to evaluate results, would be beneficial.

Funding: This work was supported by the NIHR Biomedical Research Centre at University Hospitals Bristol and Weston NHS Foundation Trust and the University of Bristol

Registration: This review was prospectively PROSPERO registered (CRD42021285019).

Word count = 299

For peer review only - http://bmjopen.bmj.com/site/about/guidelines.xhtml

Strengths and Limitations

- This review provides an up-to-date assessment of the efficacy of melatonin for prevention of postoperative delirium.
- The search was performed across a range of databases and a clinical trials registry to ensure thorough data capture.
- Studies presented in this review include a diverse range of patients, surgical subspecialties and melatonin preparations and administration regimes.
- Results of this review are limited by the heterogeneity of the included studies limits, and lack of consistency of intervention design and outcome measures.
- Language restrictions to English may have led to exclusion of additional studies.

Protocol

Published protocol has been attached a separate file. *Sewart E, Barnes J, Armstrong RA, et al. Melatonin for the prevention of postoperative delirium in older adults: a protocol for a systematic review and meta-analysis. BMJ Open 2022;12:e063405. doi:10.1136/ bmjopen-2022-063405*

For peer review only

Introduction

Post-operative delirium (POD) is a common postoperative complication, especially in older patients [1,2]. POD comprises an acute and fluctuating disturbance in attention, awareness and cognition not explained by another neurocognitive disorder [3]. It usually occurs between one to three days after surgery [3]. Although there is no single universally accepted definition. POD is associated with a number of risk factors, including pain, bleeding, polypharmacy, sedative drugs and major surgery [4–8]. POD carries a significant healthcare and economic burden and is associated with an increased length of hospital stay, increased incidence of ongoing cognitive impairment, increased care needs and increased mortality [9–11]. It is thought that around 30-40% of cases may be preventable [12,13] and strategies to prevent POD have been highlighted as an important priority for healthcare systems [14].

Melatonin, a hormone produced by the pineal gland, is known to play an important role in circadian rhythm regulation [15] and is an attractive option as an agent for the prevention of POD. It has a low-risk side-effect profile and is inexpensive. Melatonin, and melatonin receptor agonists, are used therapeutically in the treatment of sleep-wake disorders [16] and jet lag [17] and may have a role in sedation, analgesia and as an anaesthetic adjunct [18]. Furthermore, melatonin has been identified as having a potential therapeutic role in several disorders of the mind including schizophrenia, depression and POD [19–21].

A previous systematic review of six studies (conducted in 2017) examining the use of melatonin and its agonists in the prevention of POD in older adults found some evidence to suggest a beneficial effect [22]. However, these studies were mostly small, heterogeneous in their methodology, and demonstrated conflicting results. Since then, multiple studies investigating melatonin and POD have been published.

The aim of this review is to provide an up to date assessment of melatonin as an intervention for the prevention of POD in adult patients. This review will also provide a description of how melatonin has been administered (dose, duration etc.) and how POD has been studied in randomised controlled trials (RCTs).

Methods

All results from in this work are presented using the Preferred Reporting Items for Systematic Reviews and Meta-Analyses (PRISMA) guidelines and checklist [23]. We prospectively registered the review PROSPERO (CRD42021285019) and the protocol has been published [24].

Search Strategy and eligibility criteria

A search was conducted across EMBASE, Medline, CINAHL and Psychinfo for RCTs examining the effects of melatonin, or melatonin receptor agonists, in the prevention of POD. Only studies in adult patients (age 18 years or older) undergoing surgery were included. No restrictions were placed on type of surgery, the programme of drug administration (including timing, dose and additional treatments used) or on the intervention received by the comparator group. We included studies published in English between 1st January 1990 and 5th April 2022. This window was chosen in line with previous work [22], as it coincides with the development

For peer review only - http://bmjopen.bmj.com/site/about/guidelines.xhtml

of the Beers Criteria, which identified medications that may cause or worsen delirium [25]. No limits were placed on the country of study. We also hand searched the reference lists of included studies. The search strategy is detailed in Appendix 1. The search was repeated using a clinical trials registry (clinicaltrials.org) to capture ongoing, but unpublished, studies. For any studies identified, and not yet published, the study lead was contacted to request results to be shared.

Data collection

Citation management and data collection was undertaken using Covidence (Covidence, Melbourne, Australia) [26]. Titles and abstracts were independently screened by two reviewers (JB,RM). Full texts were then independently screened by the same two reviewers. All data was extracted in whole by two independent reviewers (JB, RM) using a pre-defined template. Discrepancies or disagreements at all stages were resolved by discussion between the two main reviewers. If there was still no consensus a third reviewer made the final decision (BG).

Data items

The key information gathered in the data collection form included:

1. Publication details: authors, country of study and years of study conduct and publication.
2. Participant demographics: sex, age, number, inclusion/exclusion criteria and surgery type.
3. Intervention details: drugs used, dose, timings, details of control interventions
4. Criteria and scales used for diagnosing and grading POD

The primary outcome of this review is the incidence of POD in adults undergoing surgery, with incidence of POD reflecting any recorded POD, at any timepoint. The secondary outcomes are the duration of POD and length of postoperative hospital stay.

Study risk of bias assessment

We used the Cochrane risk of bias tool, version 2, to assess for risk of bias in the estimated effect of melatonin on POD in each study across five domains [27]: The randomisation process; deviations from the intended interventions (effects of assignment to intervention and effect of adhering to intervention); missing outcome data; measurement of the outcome and selection of the reported results. The trial quality and overall risk of bias were judged as low risk, high risk or some concerns. We inspected a funnel plot and used Egger's linear regression test to detect small study effects.

Effect measures

A qualitative synthesis and narrative description of the included studies are presented alongside meta-analysis of the primary outcome. All data analysis was undertaken using Stata (StataCorp LLC, Texas, USA) [28]. Categorical data are summarised by counts and percentages, and continuous data by means and standard deviations, unless otherwise indicated. Incidence of POD is summarised using odds ratios (with associated 95% confidence intervals) for individual studies and combined using random effect meta-analysis. Between-study heterogeneity is assessed using the I^2 statistic and random-effects estimates and a fixed-effects meta-analysis is also performed as a sensitivity analysis.

Patient and public involvement

None.

For peer review only

Results

The search identified 646 studies, with 446 remaining after deduplication. From the clinical trials search 46 studies were identified, 2 were duplicated in the literature search and were excluded. 443 studies were excluded after abstract review. Of the 47 articles sought for full text review, six were from the clinical trials search. All study leads were contacted. One study was complete but unpublished and the author shared results via personal correspondence [29]. One author did not respond, and four studies were not in a position to share results (either not started recruiting or not completed data collection). After full text review, 16 articles were excluded due to the patient group (e.g. not postoperative patients) and 15 were excluded for being the wrong study type. This process is outlined in a PRISMA flowchart (Figure 1). A full list of excluded studies is included in Appendix 2.

Figure 1: PRISMA flow chart for study selection process. N = number of articles

Eleven studies including 1244 participants were eligible for inclusion in this review. Characteristics of these studies and basic participant demographics are outlined in table 1. The studies included patients across a range of surgical specialities, including general, orthopaedic and cardiothoracic surgery. One study included patients from a variety of surgical specialities [30]. Mean patient age was above 50 for all studies and several studies used a minimum age as an exclusion criterion.

For peer review only

Table 1: Characteristics of the studies and study participants

Author (year); Country	Year of study conduct	Drug (dose)(all once daily)		Timing of dosing	Duration of Therapy (days)	Study design / (blinding)	Reason for surgery	Patients N		Male patients N (%)		Age (years) Mean (SD) unless otherwise indicated		Assessment of delirium		Mean (SD) duration of surgery (minutes)	
		I	C					I	C	I	C	I	C	Scale	Timepoint	I	C
de Jonghe (2014); The Netherlands	2008-2012	Melatonin (3mg)	Placebo	Postoperative	5	RCT (double)	Hip fracture	186	192	53 (29)	62 (32)	84 (8)	83(8)	DSM-IV	Daily for 8 days post op (or until discharge)	NR	NR
Ford (2020); Australia	NR	Melatonin (3mg)	Placebo	Postoperative	7	RCT (double)	Cardiac	105	105	79 (75)	85 (81)	69 (8)	68 (8)	CAM or CAM-ICU (screening); DSM-V (scale)	Daily for 7 days if CAM positive	NR	NR
Gupta (2019); Indian	NR	Ramelteon (8mg)	Placebo	Preoperative	12 hours	RCT (double)	Mixed (requiring neuraxial anaesthesia)	50	50	35 (70)	33 (66)	69 (4)	71 (4)	CAM	Daily for 3 days	NR	NR
Hashim (unpublished); Iraq	2020	Melatonin (5mg)	No treatment	Preoperative	2 doses (night before & 90 mins pre op)	RCT (double)	NR	12	12	NR	NR	> 60	> 60	MDAS	30-, 60- and 90-minutes post-op	NR	NR
Jaiswal (2019); United States	2016-2017	Ramelteon (8mg)	Placebo	Postoperative	1 to 7	RCT (double)	Elective pulmonary thromboendarterectomy	59	28	29 (49)	29 (50)	58 (14)	56 (16)	CAM-ICU and RASS	Twice daily for 9 days (or until ICU discharge)	526 (480-540) †	510 (480-540)†
Mahrose (2021); Egypt	NR	Melatonin (5mg) + Dexmedetomidine	Dexmedetomidine (bolus + infusion)	Preoperative/ postoperative	4	RCT (NR)	Elective coronary artery bypass graft	55	55	42 (76)	41 (75)	67 (7)	66 (6)	CAM or CAM-ICU	Twice daily for 5 days (or until discharge)	NR	NR
Nickkholgh (2011); Germany	2007-2009	Melatonin (50mg/kg)	Placebo	Intraoperative	1	RCT (double)	Liver resection	25	23	17 (68)	11 (48)	59 (10)	56 (11)	NR	Up to 7 days (frequency NR)	202 (80)	212 (79)
Oh (2020); United States	2017-2019	Ramelteon (8mg)	Placebo	Postoperative	3	RCT (quadruple)	Elective primary or revision hip or knee	41	39	14 (42)	14 (37)	74 (6)	75 (5)	DSM-V	Daily for 3 days	138 (72)	162 (84)

For peer review only

							replacement										
Sultan (2010); Egypt	NR	Melatonin (5mg)	No treatment	Postoperative	2	RCT (double)	Hip arthroplasty	53	49	24 (45)	22 (45)	70 (7)	72 (6)	AMT	Daily for 3 days	127 (45)	120 (37)
Yamaguchi ^ (2014); Japan	NR	Ramelteon (8mg)	Placebo	Postoperative	4	RCT (double)	Total knee arthroplasty	22	23	NR	NR	>=70	>=70	iCDSC	Thrice daily for 4 days	NR	NR
Zadeh (2021); Iran	2018-2019	Melatonin (3mg) prolonged release	Placebo		4	RCT (double)	Coronary artery bypass graft	30	30	20 (67)	22 (73)	60 (10)	63 (8)	CAM-ICU and MDAS	Twice daily for 48 hours post extubation	NR	NR

Table 1 footnotes:
^ Abstract only
NR – not reported
I – intervention group
C – control group
mg – milligrams
kg – kilograms
RCT – randomised control trial
SD – standard deviation
CAM – Confusion Assessment Method
CAM-ICU – Confusion Assessment Method for the Intensive Care Unit
DSM – Diagnostic and statistical manual of mental disorders
ICDSC – Intensive care delirium screening checklist score
AMT – Abbreviated Mental Test
MDAS.- Memorial Delirium Assessment Scale
*Median (range)
† Median (IQR)

For peer review only

Seven studies used melatonin as the study drug, in a variety of doses (from 3mg to 50mg/kg and in one study as a long acting formulation), with one using Dexmedetomidine alongside melatonin in the intervention arm [31]. Four studies used Ramelteon (always 8mg once daily). Dosing schedule was highly variable. Three studies included preoperative dosing (two exclusively preoperative, one pre and postoperative), in one study medication was given intraoperatively, whilst the others involved exclusively postoperative dosing. In studies with *a priori* dosing regimens, dosing programmes ranged from one to seven days. In two studies patients were included if they had had at least one or three doses respectively and in two other studies the duration of treatment was dependent on the length of hospital stay. Eight different measures of delirium were used across the identified studies. One study did not report the assessment tool used [32], however all other included studies used validated assessment tools. Some studies used multiple tools for diagnosis of delirium and assessing its severity and some tools were used in multiple ways (e.g. Confusion Assessment Measure was used a screening tool in one study and a diagnostic tool in others, whilst the Memorial Delirium Assessment Scale was used a diagnostic tool in one study and a tool to grade severity in another). The time points of assessment were also highly variable (table 1).

Incidence of delirium ranged from 0% to 42% in the intervention groups and 4% to 92% in the control groups. Pooled data showed evidence of a reduced incidence of POD associated with melatonin use (OR 0.41, 95% CI 0.21 to 0.80, 11 studies, 1244 participants, p=0.01). Assuming an overall risk of POD the same as that of the combined study populations (0.23), this gives a number needed to treat of 8.40. Random-effects estimates were used as there is significant between-study heterogeneity (I^2 =71.97%) (Figure 2). However, sensitivity analysis with a fixed-effects meta-analysis gave similar results, also finding a reduced incidence of POD associated with melatonin use (OR 0.63, 95% CI 0.48-0.83, p<0.01).

Figure 2: Forest plot displaying odds ratios and 95% confidence intervals of effect of Melatonin or melatonergic agent on incidence of POD per study. Overall odds ratio generated using a random effects meta analysis.

Duration of POD and length of hospital stay were each reported by five studies (Table 2). The median durations of delirium reported by ranged from 0.7 days [33] to 3 days [34]. Mean duration of hospital stay ranged from 7 to 17 days. Due to the paucity of reported data, neither synthesis of secondary outcome data nor reporting of complications associated with treatment were performed.

For peer review only

Table 2: Study outcomes

Author (year); Country	Delirium incidence N (%)		Delirium duration (days) median (range)		Hospital stay (days) Mean (SD) unless otherwise indicated	
	I	C	I	C	I	C
de Jonghe (2014); The Netherlands	55 (30)	49 (25.5)	2 (1-3)	2 (1-3)	11 (6-14.5)*	11 (8-17)*
Ford (2020); Australia	21 (21)	21 (20)	3 (2-4)†	2 (1-3)†	8 (6-10)†	7 (6-8)†
Gupta (2019); Indian	2 (4)	6 (12)	NR	NR	NR	NR
Hashim (unpublished); Iraq	5 (42)	11 (92)	NR	NR	NR	NR
Jaiswal (2019); United States	19 (32)	22 (38)	1.0 (0.6-1.5)†	0.7 (0.4-1.2)†	12 (10-16)†	12 (10-14)†
Mahrose (2021); Egypt	6 (11)	12 (27)	1.0 (+- 0.3)	2.0 (+- 0.6)	12 (+-5)	14 (+-6)
Nickkholgh (2011); Germany	0	1 (4)	NR	NR	13.5 (+-1.5)	17 (+-2)
Oh (2020); United States	7 (21)**	3 (8)**	1.3 (+-0.5)	1.2 (+-0.5)	NR	NR
Sultan (2010); Egypt	5 (9)	16 (33)	NR	NR	NR	NR
Yamaguchi (2014); Japan	0	2 (9)	NR	NR	NR	NR
Zadeh (2021); Iran	4 (13)**	14 (47)**	NR	NR	NR	NR

For peer review only

I – Intervention group
C - Control group
NR – Not reported
SD – Standard deviation
* Median (Range)
+ Median (IQR)
** delirium incidence reported at more than one timepoint. The highest reported rate is included here.

For peer review only

All included studies used some blinding and randomisation process and a validated outcome tool(s). Six studies were considered at low risk of bias, five were rated as "some concern." This occurred due to studies lacking either a clearly pre-specified analysis plan and/or concerns regarding the blinding process. No studies were considered at high risk of bias (Appendix 3). No studies were considered at risk of financial bias. Nine studies reported no conflicts of interest. For the other studies, no author reported any support, financial or otherwise, from any source related to the study drug, nor did either study receive any private funding. A funnel plot was prepared and inspected (Appendix 4) and an Egger's and Harbord test performed. This displayed no clear evidence small study effects (Egger's p=0.128, Harbord p=0.127).

Certainty of evidence was assessed using the Grading of Recommendations Assessment, Development and Evaluation (GRADE) approach. Results are outlined in Table 3. Overall, evidence included in this review supports melatonin as effective prophylaxis for delirium with a moderate level of certainty. Recommendation was rated down due to heterogeneity/inconsistency, which could not be explained as subgroup analysis was unable to be undertaken.

Table 3: GRADE assessment of evidence certainty, Melatonin compared to placebo for prevention of post-operative delirium

Melatonin compared to placebo for prevention of post-operative delirium						
Patient or population: Adult patients undergoing surgery<br>Setting: Hospital<br>Intervention: Melatonin or melatonin receptor agonist<br>Comparison: Placebo						
Outcomes	Anticipated absolute effects		Relative effect (95% CI)	№ of participants (studies)	Certainty of the evidence (GRADE)	Comments
	Risk with placebo	Risk with melatonin				
Delirium	Study population		OR 0.41 (0.21 to 0.80)	1244 (11 RCTs)	⊕⊕⊕ MODERATE	Low risk of bias, precise, no evidence of publication bias. Rated down for inconsistency. Delirium incidence was recorded at multiple timepoints, this refers to "any delirium" during study window.
	157/606	124/638				
*The risk in the intervention group (and its 95% confidence interval) is based on the assumed risk in the comparison group and the relative effect of the intervention (and its 95% CI).<br><br>CI: Confidence interval; RR: Risk ratio; OR: Odds ratio;						
GRADE Working Group grades of evidence<br>High certainty: We are very confident that the true effect lies close to that of the estimate of the effect<br>Moderate certainty: We are moderately confident in the effect estimate: The true effect is likely to be close to the estimate of the effect, but there is a possibility that it is substantially different<br>Low certainty: Our confidence in the effect estimate is limited: The true effect may be substantially different from the estimate of the effect<br>Very low certainty: We have very little confidence in the effect estimate: The true effect is likely to be substantially different from the estimate of effect						

Discussion

For peer review only

The main findings from this systematic review are that melatonin and melatonin receptor agonists are associated with a reduced incidence of POD in adult patients undergoing surgery, whilst huge variation exists in the intervention design as well as the outcome measures employed.

The development of postoperative delirium is multi factorial. Risk factors include patient factors (e.g. pre-existing cognitive impairment, atherosclerosis), intraoperative factors (e.g. use of benzodiazepines, high stress surgery) and postoperative factors (e.g. disrupted sleep-wake cycling, pain) [35, 36]. The aetiology is thought to include a direct neuroinflammatory process, accompanied by disruption in various neurotransmitter pathways [36, 37]. Melatonin may act to reduce POD both by ensuring, and restoring, normal sleep-wake cycling along with exerting a direct anti-inflammatory action [38], involving free radical scavenging, immune modulation and direct neuroprotection [39].

Despite the rationale for melatonin preventing POD, a recent RCT found no impact of melatonin on incidence of POD in adult intensive care (ICU) patients [40]. However, this study assessed only critically unwell patients who started taking melatonin treatment after once they had been admitted to intensive care. This therefore does not rule out potential benefit of melatonin in different patient groups and in different treatment regimens. The results from our review concur with the findings from two previous, smaller reviews on postoperative patient populations [22, 41]. These reviews both suggest melatonin as an effective prevention for POD, but are limited by small numbers of heterogenous studies. Since both of these reviews, there has been further interest in the effects of melatonin on postoperative delirium, allowing us to include multiple additional RCTs in this review. The reduction of POD seen with melatonin in our work is also in keeping with the results from a mixed patient review, which concluded that "melatonin/ramelteon are associated with reduction in delirium… however this effect seems confined to surgical and ICU patients [42]."

Multiple databases were searched, using a standardised search, along with hand searching of references and a registry search, ensuring good capture of includable studies. No included studies were at high risk of bias and those included captured a wide variety of patient groups and surgical specialities. Alongside qualitative analysis of the available evidence this study also adds quantitative evaluation of synthesised data on the effect of melatonin on preventing POD.

Our review is limited by the heterogeneity of the published trials of melatonin for the prevention of POD. The study interventions were highly variable, including populations, drugs used, dosing and administration regimens. The outcome measures used varied widely across studies, and there was little consistency in when assessments were undertaken, or how frequently. Given delirium has a fluctuant nature, this may have contributed to under-reporting of delirium in several trials. The lack of consistency of either intervention or outcome measure also makes it challenging to conclude what an optimal melatonin dosing schedule would look like, and evaluation of this would be a valuable area for further study. This heterogeneity, along with the small number of included studies, also made it impractical to perform a sensitivity analysis of results, further limiting the strength of our results. Most studies excluded patients with pre-existing cognitive impairment and those on known psychoactive medications. These patients represent some of the highest risk of POD and would therefore arguably be some of those most likely to benefit from POD reduction strategies. Furthermore, the interventions used in the included studies may not reflect the

For peer review only

likely use of melatonin in practice, limiting the external validity of these results. It is likely that future POD prevention strategies will involve multi-faceted care bundles, rather than single interventions (e.g. non pharmaceutical sleep hygiene strategies, pro-active pain management, avoidance of other exacerbating medications etc. along with any pharmaceutical agent(s)). A final limitation was that we were unable to undertake subgroup analyses due to patient numbers. This would be an area for future study, particularly in the cardiac surgical population, who, due to the nature of the surgery, anaesthesia and recovery, are a group at particularly high risk of POD [43, 44].

In conclusion, melatonin has both biologic plausibility and clinical evidence to support it as a treatment to prevent POD. However, the optimum protocol for melatonin use is yet to be determined. A consensus on how best to deliver the intervention and evaluate results is needed before further, adequately-powered clinical evaluation can be performed to properly analyse melatonin as a preventative intervention for delirium. Clinical applicability of findings from future work may be enhanced by focusing on those at highest risk of POD.

Contributorship

JB and ES are joint first authors and RM and BG are joint final authors.
All authors have contributed fully to the concept and design of the review for which this protocol has been written. JB, ES, BG and RM wrote and reviewed the manuscript before submission. RA supervised the meta-analysis. MP, RA and RJH reviewed and edited the manuscript before submission. RM is the guarantor of the review.

Funding Statement

This work was supported by the NIHR Biomedical Research Centre at University Hospitals Bristol and Weston NHS Foundation Trust and the University of Bristol. RA and ES are NIHR Academic Clinical Fellows. The views expressed in this publication are those of the author(s) and not necessarily those of the NHS, the National Institute for Health Research, the Department of Health and Social Care.

Competing Interests

None declared.

Ethics and dissemination

No ethical approval is required for this manuscript. Findings will be presented through conferences and peer reviewed publication.

Data Sharing

No data available. As a systematic review and meta-analysis, all data used is from other articles. No original research data was generated.

Acknowledgements

We thank Professor Jonathan Sterne, professor of medical statistics and epidemiology, for his assistance with assessment of potential reporting bias and Sarah Rudd, Knowledge Specialist, for her assistance with the literature search.

Word count: 3912 (incl. tables, excl. abstract)

For peer review only

For peer review only

For peer review only - http://bmjopen.bmj.com/site/about/guidelines.xhtml

References

1. Inouye SK. Delirium in older persons. *The New England Journal of Medicine* 2006; **354**: 1157–65.

2. Inouye SK, Westendorp RGJ, Saczynski JS. Delirium in elderly people. *Lancet (London, England)* 2014; **383**: 911–22.

3. American Psychiatric Association. *Diagnostic and Statistical Manual of Mental Disorders: DSM-5.*, 5th edition. Washington, D.C.: American Psychiatric Publishing., 2013.

4. Inouye SK, Charpentier PA. Precipitating factors for delirium in hospitalized elderly persons. Predictive model and interrelationship with baseline vulnerability. *JAMA* 1996; **275**: 852–7.

5. Vaurio LE, Sands LP, Wang Y, Mullen EA, Leung JM. Postoperative delirium: the importance of pain and pain management. *Anesthesia and Analgesia* 2006; **102**: 1267–73.

6. Oldroyd C, Scholz AFM, Hinchliffe RJ, McCarthy K, Hewitt J, Quinn TJ. A systematic review and meta-analysis of factors for delirium in vascular surgical patients. *Journal of Vascular Surgery* 2017; **66**: 1269-1279.e9.

7. Galyfos GC, Geropapas GE, Sianou A, Sigala F, Filis K. Risk factors for postoperative delirium in patients undergoing vascular surgery. *Journal of Vascular Surgery* 2017; **66**: 937–46.

8. Bilotta F, Lauretta MP, Borozdina A, Mizikov VM, Rosa G. Postoperative delirium: risk factors, diagnosis and perioperative care. *Minerva Anestesiologica* 2013; **79**: 1066–76.

9. Leslie DL, Zhang Y, Holford TR, Bogardus ST, Leo-Summers LS, Inouye SK. Premature death associated with delirium at 1-year follow-up. *Archives of Internal Medicine* 2005; **165**: 1657–62.

10. Buurman BM, Hoogerduijn JG, de Haan RJ et al. Geriatric conditions in acutely hospitalized older patients: prevalence and one-year survival and functional decline. *PloS One* 2011; **6**: e26951.

11. Pitkala KH, Laurila JV, Strandberg TE, Tilvis RS. Prognostic significance of delirium in frail older people. *Dementia and Geriatric Cognitive Disorders* 2005; **19**: 158–63.

12. Inouye SK, Bogardus ST, Charpentier PA et al. A multicomponent intervention to prevent delirium in hospitalized older patients. *The New England Journal of Medicine* 1999; **340**: 669–76.

13. Marcantonio ER, Flacker JM, Wright RJ, Resnick NM. Reducing delirium after hip fracture: a randomized trial. *Journal of the American Geriatrics Society* 2001; **49**: 516–22.

14. O'Mahony R, Murthy L, Akunne A, Young J. Synopsis of the National Institute for Health and Clinical Excellence Guideline for Prevention of Delirium. *Annals of Internal Medicine* 2011; **154**: 746–51.

For peer review only

15. Zisapel N. New perspectives on the role of melatonin in human sleep, circadian rhythms and their regulation. *British Journal of Pharmacology* 2018; **175**: 3190–9.

16. Buscemi N, Vandermeer B, Hooton N et al. Efficacy and safety of exogenous melatonin for secondary sleep disorders and sleep disorders accompanying sleep restriction: meta-analysis. *BMJ (Clinical research ed.)* 2006; **332**: 385–93.

17. Petrie K, Conaglen JV, Thompson L, Chamberlain K. Effect of melatonin on jet lag after long haul flights. *BMJ : British Medical Journal* 1989; **298**: 705–7.

18. Procaccini D, Lobner K, Azamfirei R, Kudchadkar SR. Melatonin for anaesthetic indications in paediatric patients: a systematic review. *Anaesthesia* 2021; **76**: 837–49.

19. Morera-Fumero AL, Abreu-Gonzalez P. Role of melatonin in schizophrenia. *International Journal of Molecular Sciences* 2013; **14**: 9037–50.

20. Cardinali DP, Srinivasan V, Brzezinski A, Brown GM. Melatonin and its analogs in insomnia and depression. *Journal of Pineal Research* 2012; **52**: 365–75.

21. Scholtens RM, de Rooij SEJA, Vellekoop AE, Vrouenraets BC, van Munster BC. Preoperative CSF Melatonin Concentrations and the Occurrence of Delirium in Older Hip Fracture Patients: A Preliminary Study. *PloS One* 2016; **11**: e0167621.

22. Campbell AM, Axon DR, Martin JR, Slack MK, Mollon L, Lee JK. Melatonin for the prevention of postoperative delirium in older adults: a systematic review and meta-analysis. *BMC Geriatrics* 2019; **19**: 272.

23. Page MJ, McKenzie JE, Bossuyt PM et al. The PRISMA 2020 statement: an updated guideline for reporting systematic reviews. *BMJ* 2021; **372**: n71.

24. Sewart E, Barnes J, Armstrong RA et al. Melatonin for the prevention of postoperative delirium in older adults: a protocol for a systematic review and meta-analysis. *BMJ Open* 2022; **12**: e063405.

25. Beers MH, Ouslander JG, Rollingher I, Reuben DB, Brooks J, Beck JC. Explicit criteria for determining inappropriate medication use in nursing home residents. UCLA Division of Geriatric Medicine. *Archives of Internal Medicine* 1991; **151**: 1825–32.

26. Veritas Health Innovation. Covidence systematic review software.https://www.covidence.org/.

27. Sterne JAC, Savović J, Page MJ et al. RoB 2: a revised tool for assessing risk of bias in randomised trials. *BMJ* 2019; **366**: l4898.

28. StataCorp. Stata Statistical Software: Release 17. 2021.

29. Hashim H. The efficacy of oral Melatonin in preventing Postoperative Delirium for patients undergoing orthopedic surgery under general anesthesia: A randomized controlled trial. *Unpublished*2022.

For peer review only

30. Gupta P, Verma R, Kohli M, Shukla N, Kannaujia S. The Effect of Ramelteon on Postoperative Delirium in Elderly Patients: A Randomised Double-Blind Study. *JOURNAL OF CLINICAL AND DIAGNOSTIC RESEARCH*2019; doi 10.7860/jcdr/2019/42635.13384.

31. Mahrose R, ElSerwi H, Maurice A, Elsersi M. Postoperative delirium after coronary artery bypass graft surgery: Dexmedetomidine infusion alone or with the addition of oral melatonin. *Egyptian Journal of Anaesthesia* 2021; **37**: 62–8.

32. Nickkholgh A, Schneider H, Sobirey M et al. The use of high-dose melatonin in liver resection is safe: first clinical experience. *Journal of Pineal Research* 2011; **50**: 381–8.

33. Jaiswal SJ, Vyas AD, Heisel AJ et al. Ramelteon for Prevention of Postoperative Delirium: A Randomized Controlled Trial in Patients Undergoing Elective Pulmonary Thromboendarterectomy. *Critical Care Medicine* 2019; **47**: 1751–8.

34. Ford AH, Flicker L, Kelly R et al. The Healthy Heart-Mind Trial: Randomized Controlled Trial of Melatonin for Prevention of Delirium. *Journal of the American Geriatrics Society* 2020; **68**: 112–9.

35. Bagri AS, Rico A, Ruiz JG. Evaluation and management of the elderly patient at risk for postoperative delirium. *Clin Geriatr Med.* 2008; **24**(4): 667–686.

36. Whitlock EL, Vannucci A, Avidan MS. Postoperative delirium. *Minerva Anestesiol*. 2011; **77**(4): 448-56.

37. Vacas S, Degos V, Feng X, *et al* . The neuroinflammatory response of postoperative cognitive decline. *Br Med Bull* 2013; **106**: 161–167

38. Carrillo-Vico A, Lardone PJ, Alvarez-Sánchez N, et al. Melatonin: buffering the immune system. *Int J Mol Sci.* 2013; 14; 8638–8683.

39. Kilic E, Kilic U, Bacigaluppi M, et al. Delayed melatonin administration promotes neuronal survival, neurogenesis and motor recovery, and attenuates hyperactivity and anxiety after mild focal cerebral ischemia in mice. *J Pineal Res* 2008; 45: 142–148.

40. Wibrow B, Martinez FE, Myers E et al. Prophylactic melatonin for delirium in intensive care (Pro-MEDIC): a randomized controlled trial. *Intensive Care Medicine* 2022; **48**: 414–25.

41. Han Y, Wu J, Qin Z, et al. Melatonin and its analogues for the prevention of postoperative delirium: A systematic review and meta-analysis. *J Pineal Res.* 2020; 68**(4);**

42. Khaing K, Nair BR. Melatonin for delirium prevention in hospitalized patients: A systematic review and meta-analysis. *J Psychiatr Res*. 2021; 133; 181-190.

43. Smith LW, Dimsdale JE. Postcardiotomy delirium: Conclusions after 25 years? *Am J Psychiatry.* 1989; 146: 452–458

44. Blachy PH, Starr A. Post-cardiotomy delirium. *Am JPsychiatry.* 1964;121:371–5

For peer review only

PRISMA 2020 flow diagram for new systematic reviews which included searches of databases and registers only
MELATONIN FOR THE PREVENTION OF POST OPERATIVE DELIRIUM

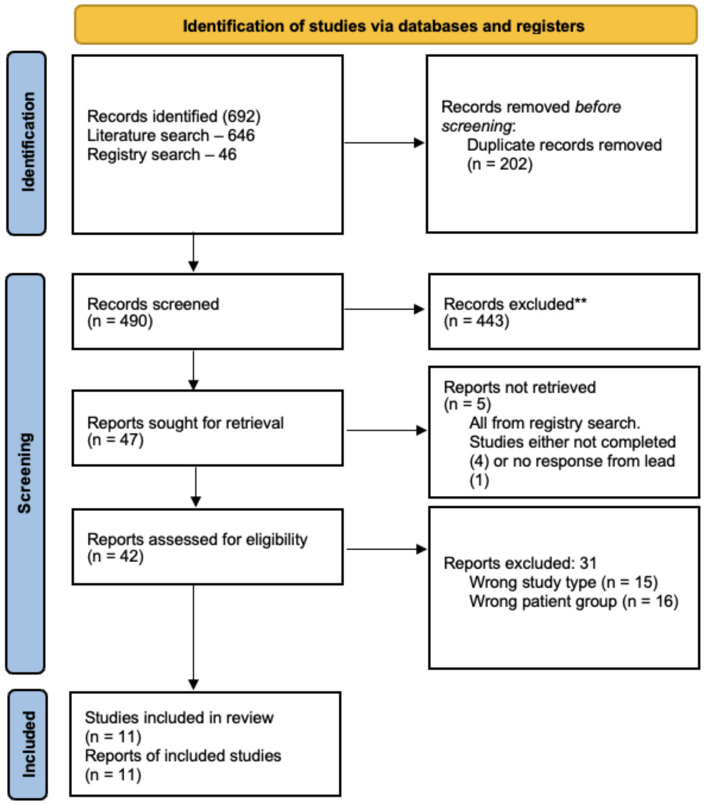

*Consider, if feasible to do so, reporting the number of records identified from each database or register searched (rather than the total number across all databases/registers).

**If automation tools were used, indicate how many records were excluded by a human and how many were excluded by automation tools.

Figure 1: PRISMA flow chart for study selection process. N = number of articles

148x167mm (144 x 144 DPI)

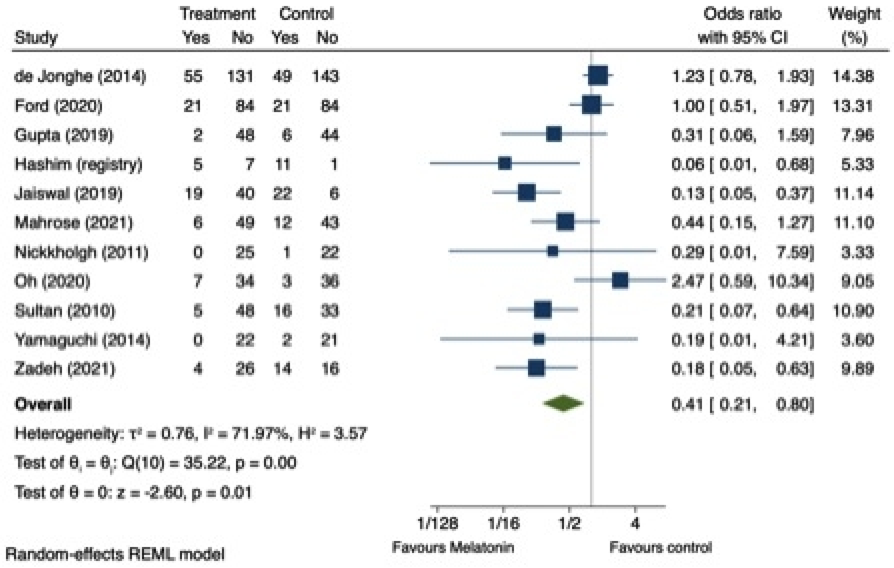

Figure 2: Forest plot displaying odds ratios and 95% confidence intervals of effect of Melatonin or melatonergic agent on incidence of POD per study. Overall odds ratio generated using a random effects meta analysis.

159x102mm (72 x 72 DPI)

Appendix 1: Search Strategy

[melatonin(MH) OR ramelteon(TW) OR rozerem(TW) OR “melatonin agonist*”(TW) OR “N-Acetyl-5-methoxytryptamine”(TW) OR melatonin*(TW) OR Melatoniin*(TW) OR circadin(TW) OR “HT 903”(TW) OR melapure(TW) OR armonia(TW) OR melamil(TW) OR benedorm(TW) OR “BP 2013”(TW) OR BP2013 OR JL5DK93RCL(TW)]

AND

[delirium(MH) OR “emergence delirium”(MH), OR delirium(TW) OR “perioperative delirium”(TW) OR “postoperative delirium”(TW) OR “organic brain syndrome”(TW) OR “acute confusion”(TW)]

Dates: 1st January 1990 – 5th April 2022

Other limits: Nil

For peer review only

CINAHL Search Strategy

#	Query
**S24**	S16 AND S22
**S23**	S16 AND S22
**S22**	S17 OR S18 OR S19 OR S20 OR S21
**S21**	((postoperative or perioperative) N1 delirium)
**S20**	"acute confusion"
**S19**	"organic brain syndrome"
**S18**	Delirium
**S17**	(MH "Delirium")
**S16**	S1 OR S2 OR S3 OR S4 OR S5 OR S6 OR S7 OR S8 OR S9 OR S10 OR S11 OR S12 OR S13 OR S14 OR S15
**S15**	"JL5DK93RCL"
**S14**	"bp2013"
**S13**	"bp 2013"
**S12**	benidorm
**S11**	melamil
**S10**	armenia
**S9**	melapure
**S8**	"ht 903"
**S7**	circadian
**S6**	melatoniin*
**S5**	"n-acetyl-5-methoxytryptamine"
**S4**	rozerem
**S3**	ramelteon
**S2**	melatonin*
**S1**	(MM "Melatonin")

Dates – 1/1/1990 – 5/4/2022

For peer review only

Ovid Medline All and Embase

1.	**exp Melatonin/**
2.	melatonin*.mp.
3.	ramelteon.mp.
4.	rozerem.mp.
5.	"n-acetyl-5-methoxytryptamine".mp.
6.	melatoniin*.mp.
7.	circadian.mp.
8.	"ht 903".mp.
9.	melapure.mp.
10.	armonia.mp.
11.	melamil.mp.
12.	benedorm.mp.
13.	"bp 2013".mp.
14.	"BP2013".mp.
15.	"JL5DK93RCL".mp.
16.	1 or 2 or 3 or 4 or 5 or 6 or 7 or 8 or 9 or 10 or 11 or 12 or 13 or 14 or 15
17.	Delirium/
18.	Emergence Delirium/
19.	delirium.mp.
20.	"organic brain syndrome".mp.
21.	"acute confusion".mp.
22.	((postoperative or perioperative) adj1 delirium).mp.
23.	17 or 18 or 19 or 20 or 21 or 22
24.	16 and 23
25.	24
26.	limit 25 to yr="1990 -Current"
27.	26 use medall
28.	melatonin/
29.	melatonin*.mp.
30.	ramelteon.mp.
31.	rozerem.mp.
32.	"n-acetyl-5-methoxytryptamine".mp.
33.	melatoniin*.mp.
34.	circadian.mp.
35.	"ht 903".mp.
36.	melapure.mp.
37.	armonia.mp.
38.	melamil.mp.
39.	benedorm.mp.
40.	"bp 2013".mp.
41.	"BP2013".mp.
42.	"JL5DK93RCL".mp.
43.	28 or 29 or 30 or 31 or 32 or 33 or 34 or 35 or 36 or 37 or 38 or 39 or 40 or 41 or 42
44.	exp delirium/
45.	delirium.mp.
46.	"organic brain syndrome".mp.
47.	"acute confusion".mp.
48.	((postoperative or perioperative) adj1 delirium).mp.
49.	44 or 45 or 46 or 47 or 48
50.	43 and 49
51.	50
52.	limit 51 to yr="1990 -Current"
53.	52 use oemezd
54.	27 or 53
55.	54

For peer review only - http://bmjopen.bmj.com/site/about/guidelines.xhtml

Psychinfo: **Search Strategy**

Set#	Searched for
S1	MAINSUBJECT.EXACT("Melatonin")
S2	melatonin*
S3	ramelteon
S4	rozerem
S5	"n-acetyl-5-methoxytryptamine"
S6	melatoniin*
S7	circadian
S8	"ht 903"
S9	melapure
S10	armonia
S11	melamil
S12	benedorm
S13	"bp 2013"
S14	"bp2013"
S15	"JL5DK93RCL"
S16	OR(1:15)
S17	MAINSUBJECT.EXACT.EXPLODE("Delirium")
S18	Delirium
S19	"organic brain syndrome"
S20	"acute confusion"
S21	((postoperative or perioperative) N/1 delirium)
S22	OR(17:21)
S23	16 AND 22
S24	23 (limited to 1990-current)

For peer review only

Trials Registry

CONDITION: DELERIUM
OTHER: MELATONIN OR RAMELTEON OR ROZEREM OR n-acetyl-5-methoxytryptamine OR HT 903 OR MELAPURE OR ARMONIA OR MELAMIL OR BENEDORM OR JL5DK93RCL
STUDY TYPE: ALL
STUDY RESULTS: ALL

For peer review only

Appendix 2: List of studies excluded after full text review.

Study	Primary Reason for exclusion
Lam K et al. Effectiveness of Melatonin for the Prevention of Intensive Care Unit Delirium. *Pharmacotherapy*. 2019.	Wrong population
Bellapart J et al. Effect of Exogenous Melatonin Administration in Critically Ill Patients on Delirium and Sleep: A Randomized Controlled Trial. *Critical Care Research and Practice*. 2020	Wrong population
Bouajram R et al. Efficacy of sleep medication administration and impact on delirium in critically ill patients. *Critical Care Medicine*. 2021	Wrong population
Gandolfi J et al. The Effects of Melatonin Supplementation on Sleep Quality and Assessment of the Serum Melatonin in ICU Patients: A Randomized Controlled Trial. *Critical Care Medicine*. 2020	Wrong population
Jung S et al. Effectiveness of melatonin for the prevention of icu delirium: could it be dose-dependent? *Critical Care Medicine*. 2021	Wrong study design
Villa C et al. Enteral versus intravenous approach for the sedation of critically ill patients: A randomized and controlled trial. *Critical Care*. 2019	Wrong population
Daniels L at al. Pharmacologic Treatment of Intensive Care Unit Delirium and the Impact on Duration of Delirium, Length of Intensive Care Unit Stay, Length of Hospitalization, and 28-Day Mortality. *Mayo Clinic Proceedings*. 2018	Wrong population
Romero N et al. Evaluation of Delirium in Critically Ill Patients Prescribed Melatonin or Ramelteon. *Annals of Pharmacotherapy*. 2021	Wrong population
Romero N at al. Evaluation of melatonin and ramelteon for delirium prevention in critically ill patients. *Critical Care Medicine*. 2019.	Wrong population
Hamidi A et al. Documented sleep quality and scheduled sleep aid medication response in critically ill adults. *Critical Care Medicine*. 2019	Wrong population
Baumgartner L. Melatonin for the prevention of intensive care unit (ICU) delirium. *Critical Care Medicine*. 2018.	Wrong study design
Kikuchi N et al. Assessment of the relationship between hypnotics and delirium using the japanese adverse drug event report (JADER) database. *Yaguka Zasshi*. 2018	Wrong population
Ryo M et al. Efficacy of Ramelteon for delirium after lung cancer surgery. *Interactive Cardiovascular and Thoracic Surgery*. 2017	Wrong study design
Abbasi S et al. Potential role of exogenous melatonin supplement in delirium prevention in critically ill patients: A double-blind randomized pilot study. *Iranian Journal of Pharaceutical Research*. 2018	Wrong population
Forte M et al. The use of melatonin to prevent delirium in the critically ill elderly. *American Journal of Respiratory and Critical Care* Preventive role of ramelteon and suvorexant for postoperative delirium after pharyngolaryngectomy with esophagectomy *Medicine*. 2019	Wrong population
Kawada T. Preventive role of ramelteon and suvorexant for postoperative delirium after pharyngolaryngectomy with esophagectomy. *Esophagus*. 2018	Wrong study design
Hatta K et al. Real-World Effectiveness of Ramelteon and Suvorexant for Delirium Prevention in 948 Patients With Delirium Risk Factors. *The Journal of Clinical Psychiatry*. 2019	Wrong population

For peer review only - http://bmjopen.bmj.com/site/about/guidelines.xhtml

Wibrow B et al. Statistical analysis plan for the Prophylactic Melatonin for Delirium in Intensive Care (ProMEDIC): a randomised controlled trial. *Trials*. 2021	Wrong study design
Bobb E et al. Evaluation of melatonin on delirium prevention in the intensive care unit. *Critical Care Medicine*. 2019	Wrong study design
Fosnight S et al. Melatonin effect on incident delirium in hospitalized patients. *Journal of the American Geriatrics Society*. 2018.	Wrong population
Jouin G et al. Delirium in elderly patients: Successful use of melatonin. *International Journal of Clinical Pharmacy*. 2017	Wrong study design
Shi Y. Effects of Melatonin on Postoperative Delirium After PCI in Elderly Patients: A Randomized, Single-Center, Double-Blind, Placebo-Controlled Trial. *The Heart Surgery Forum*. 2021	Wrong population
Arizumi F et al. Efficacy of intervention for prevention of postoperative delirium after spine surgery. *Spine Surgery and Related Research*. 2021	Wrong study design
Xue ZJ et al. Assessing Preventative Effect of Ramelteon on Postoperative Delirium in Older Patients: Methodology Is Important. *American Journal of Geriatric Psychiatry*. 2021.	Wrong study design
Mu S et al. Relationship among melatonin, postoperative delirium, and postoperative cognitive dysfunction. *Annals of Palliative Medicine*. 2021	Wrong study design
Hokuto D et al. Preventative effects of ramelteon against postoperative delirium after elective liver surgery. *PLoS ONE*. 2020	Wrong study design
Shinsaku H et al. A phase II study of ramelteon for the prevention of postoperative delirium in elderly patients undergoing gastrectomy. *Surgery Today*. 2020	Wrong study design
Lopez C et al. Ramelteon for decreasing delirium in surgical intensive care unit patients. *Clinical Medicine Insights*. 2020.	Wrong population
Tsubosa Y et al. Postoperative delirium after pharyngolaryngectomy with esophagectomy: a role for ramelteon and suvorexant. *Esophagus*. 2017	Wrong study design
Welch S et al. Factors associated with delirium in surgical intensive care unit patients treated with supplemental melatonin: A case-cohort study. *Clinical neuropharmacology*. 2019	Wrong study design

Appendix 3: Risk of Bias Assessment

Risk of Bias (ROB) assessment

Trial	Ra.	As.	Ad.	Mi.	Me.	S.	Overall ROB
De Jonghe 2014	+	+	+	+	+	+	+
Ford 2020	+	+	+	+	+	+	+
Hashib *unpublished*	+	+	+	+	+	!	!
Gupta 2019	+	+	+	+	+	+	+
Jaiswal 2019	+	+	+	+	+	+	+
Mahrose 2021	+	!	!	+	!	+	!
Nickholgh 2011	+	+	+	+	+	+	+
Oh 2020	+	+	+	+	!	+	!
Sultan 2010	+	+	+	+	!	!	!
Yamaguchi 2014	+	!	+	+	+	+	!
Zadeh 2021	+	+	+	+	+	+	+

\+ Low
! Some concern
\- High

Ra. Randomisation process
As. Assignment to intervention
Ad. Adherence to intervention
Mi. Missing outcome data
Me. Measurement of outcome
S. Selection of results

view only

Appendix 4: Funnel plot

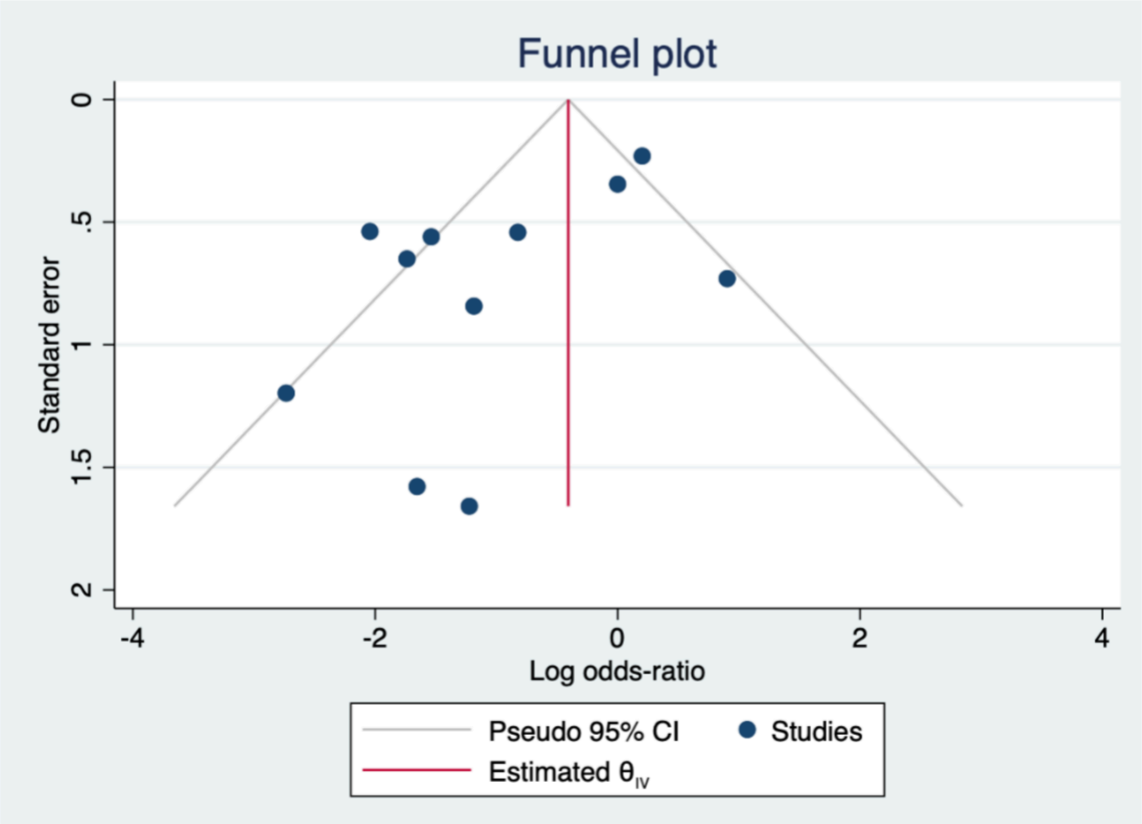

view only
